# Supplementary material for: Low-value MRI of the knee in Norway: a register-based study to identify the proportion of potentially low-value MRIs and estimate the related costs
Source: BMJ Open. 2024 Mar 13;14(3):e081860. doi: 10.1136/bmjopen-2023-081860 (PMC10941154; doi:10.1136/bmjopen-2023-081860)
Supplement: Supplementary data [file bmjopen-2023-081860supp001.pdf]

Queries made by Department for Medical Reimbursement, Norwegian Directorate of Health

Lists pasientID and dates for MR Kne, point 1

```
select e.pasient_identifikasjon, to_char(e.datotid, 'YYYY-MM-DD') as dato
from odb_ncrpkode partition (y2021) n, odb_enkeltregning partition (y2021) e, odb_innsending i
where i.fagomraade_kode IN ('PO','LR') and
N.undersokelseskode in ('SNG0AG') and
i.innsending_status_k = 'godkjent' and
e.enkeltregning_status_k in ('godkjent', 'manuelt_godkjent') and
e.datotid >= '01.07.2021' and
e.datotid < '01.01.2022' and
n.enkeltregning_id = e.enkeltregning_id and
i.innsending_id = e.innsending_id
group by e.pasient_identifikasjon, to_char(e.datotid, 'YYYY-MM-DD')
order by 1,2;
```

Lists patients registered with selected diagnosis the last six moths after first MRI of the knee (SNG0AG), and removes the details to list relevant patients

```
select r.pasient_identifikasjon, r.forste_behandling, d.diagnose_kode, to_char(MIN(e.datotid),'dd.mm.yyyy') as
dato_diagnose from
(
select e.pasient_identifikasjon, MIN(e.datotid) as forste_behandling
from odb_ncrpkode partition (y2021) n, odb_enkeltregning partition (y2021) e, odb_innsending i
where 1=1
and i.innsending_id = e.innsending_id
and n.enkeltregning_id = e.enkeltregning_id
and i.fagomraade_kode IN ('PO','LR')
and n.undersokelseskode in ('SNG0AG')
and e.enkeltregning_status_k in ('godkjent', 'manuelt_godkjent')
and e.datotid >= '01.07.2021'
and e.datotid < '01.01.2022'
group by e.pasient_identifikasjon
) r, odb_enkeltregning e, odb_diagnose d, odb_innsending i where 1=1
and e.innsending_id = i.innsending_id
and e.enkeltregning_id = d.enkeltregning_id
and r.pasient_identifikasjon = e.pasient_identifikasjon
and i.fagomraade_kode IN('LE','PO')
and e.datotid > r.forste_behandling
and e.datotid < add_months(r.forste_behandling,6)
and d.diagnose_kode
IN('M220','M221','M222','M223','M224','M228','M229','M230','M231','M232','M233','M234','M235','M236','M238','M2
39','M660','M705','M712','M924','S832')
group by r.pasient_identifikasjon, r.forste_behandling, d.diagnose_kode;
```

Lists patients registered with specific diagnosis the first six months after the MRI of the knee – point 2 shows public/private distribution

```
select r.pasient_identifikasjon, r.fagomraade_kode from
(
select e.pasient_identifikasjon, i.fagomraade_kode, MIN(e.datotid) as forste_behandling
from odb_ncrpkode partition (y2021) n, odb_enkeltregning partition (y2021) e, odb_innsending i
where 1=1
and i.innsending_id = e.innsending_id
and n.enkeltregning_id = e.enkeltregning_id
and i.fagomraade_kode IN ('PO','LR')
and n.undersokelseskode in ('SNG0AG')
and e.enkeltregning_status_k in ('godkjent', 'manuelt_godkjent')
and e.datotid >= '01.07.2021'
and e.datotid < '01.01.2022'
group by e.pasient_identifikasjon, i.fagomraade_kode
) r, odb_enkeltregning e, odb_diagnose d, odb_innsending i where 1=1
and e.innsending_id = i.innsending_id
and e.enkeltregning_id = d.enkeltregning_id
and r.pasient_identifikasjon = e.pasient_identifikasjon
and i.fagomraade_kode IN('LE','PO')
and e.datotid > r.forste_behandling
and e.datotid < add_months(r.forste_behandling,6)
```

```
and d.diagnose_kode
IN('M220','M221','M222','M223','M224','M228','M229','M230','M231','M232','M233','M234','M235','M236','M238','M2
39','M660','M705','M712','M924','S832')
group by r.pasient_identifikasjon, r.fagomraade_kode;
```

Lists patients registered with specified diagnosis the six first months after MRI – point 3 shows shows those registered with a doctor/physiotherapist 6 months before the first SNG0AG, remove the details of the number of patients

```
select r.pasient_identifikasjon from
(
select e.pasient_identifikasjon, MIN(e.datotid) as forste_behandling
from odb_ncrpkode partition (y2021) n, odb_enkeltregning partition (y2021) e, odb_innsending i
where 1=1
and i.innsending_id = e.innsending_id
and n.enkeltregning_id = e.enkeltregning_id
and i.fagomraade_kode IN ('PO','LR')
and n.undersokelseskode in ('SNG0AG')
and e.enkeltregning_status_k in ('godkjent', 'manuelt_godkjent')
and e.datotid >= '01.07.2021'
and e.datotid < '01.01.2022'
group by e.pasient_identifikasjon
) r, odb_enkeltregning e, odb_diagnose d, odb_innsending i where 1=1
and e.innsending_id = i.innsending_id
and e.enkeltregning_id = d.enkeltregning_id
and r.pasient_identifikasjon = e.pasient_identifikasjon
and i.fagomraade_kode IN('LE')
and i.praksis_refusjonsgrunnlag in ('Fastlege','Fastlønnet','Turnuslege fastlønnet')
--and i.fagomraade_kode IN('LE','FY')
--and i.praksis_refusjonsgrunnlag not in ('Legevakt','Legevakt kommunal')
and e.datotid < r.forste_behandling
and e.datotid > add_months(r.forste_behandling,-6)
and d.diagnose_tabell in ('ICPC-2','ICPC-2B')
and (d.diagnose_kode like ('L78%') or
d.diagnose_kode like ('L96%'))
group by r.pasient_identifikasjon;group by r.pasient_identifikasjon, r.forste_behandling, d.diagnose_kode;
```

Lists the patients who are registered with SNG0AG, but who are not registered with any of the selected diagnostic codes six months before the first date with SNG0AG, point 3

```
select r.pasient_identifikasjon, r.forste_behandling from
(
select e.pasient_identifikasjon, MIN(e.datotid) as forste_behandling
from odb_ncrpkode partition (y2021) n, odb_enkeltregning partition (y2021) e
--, odb_innsending i
where 1=1
--and i.innsending_id = e.innsending_id
and n.enkeltregning_id = e.enkeltregning_id
--and i.fagomraade_kode IN ('PO','LR')
and e.enkeltregning_status_k in ('godkjent', 'manuelt_godkjent')
and n.undersokelseskode in ('SNG0AG')
and e.datotid >= '01.07.2021'
and e.datotid < '01.01.2022'
group by e.pasient_identifikasjon
) r where 1=1
and not exists(
select 0 from odb_enkeltregning partition (y2021) e, odb_diagnose partition (y2021) d, odb_innsending i where
1=1
and e.innsending_id = i.innsending_id
and e.enkeltregning_id = d.enkeltregning_id
and r.pasient_identifikasjon = e.pasient_identifikasjon
and i.fagomraade_kode IN('LE')
and i.praksis_refusjonsgrunnlag in ('Fastlege','Fastlønnet','Turnuslege fastlønnet')
--and i.fagomraade_kode IN('LE','FY')
--and i.praksis_refusjonsgrunnlag not in ('Legevakt','Legevakt kommunal')
and e.datotid < r.forste_behandling
and e.datotid > add_months(r.forste_behandling,-6)
and d.diagnose_tabell in ('ICPC-2','ICPC-2B')
and (d.diagnose_kode like ('L78%') or d.diagnose_kode like ('L96%'))
```

```
);
```

Lists patients registered with SNG0AG and registered with selected NCRP codes up to six months before the first date with SNG0AG, point 4

```
select r.pasient_identifikasjon from
(
select e.pasient_identifikasjon, MIN(e.datotid) as forste_behandling
from odb_ncrpkode partition (y2021) n, odb_enkeltregning partition (y2021) e, odb_innsending i
where 1=1
and i.innsending_id = e.innsending_id
and n.enkeltregning_id = e.enkeltregning_id
and i.fagomraade_kode IN ('PO','LR')
and n.undersokelseskode in ('SNG0AG')
and e.enkeltregning_status_k in ('godkjent', 'manuelt_godkjent')
and e.datotid >= '01.07.2021'
and e.datotid < '01.01.2022'
group by e.pasient_identifikasjon
) r, odb_enkeltregning e, odb_ncrpkode partition (y2021) n, odb_innsending i where 1=1
and e.innsending_id = i.innsending_id
and e.enkeltregning_id = n.enkeltregning_id
and r.pasient_identifikasjon = e.pasient_identifikasjon
and i.fagomraade_kode IN('PO','LR')
and e.datotid < r.forste_behandling
and e.datotid > add_months(r.forste_behandling,-6)
and n.undersokelseskode in ('SNG0AA','SNG0AD','SNG0AK')
group by r.pasient_identifikasjon;
```

How many in the sample above are not included in the UU1 sample, item 4

```
select count (distinct p.fnr)
from TMP_VH_23_HOFMANN_SML_MED_UU1 p where 1=1
and not exists(select 0 from TMP_VH_23_HOFMANN_UU1 r where 1=1
and p.fnr = r.fnr);
```

How many in the main sample have M17, but not SNG0AA

```
select r.pasient_identifikasjon from
(
select e.pasient_identifikasjon, i.fagomraade_kode, MIN(e.datotid) as forste_behandling
from odb_ncrpkode partition(y2021) n, odb_enkeltregning partition(y2021) e, odb_innsending i
where 1=1
and i.innsending_id = e.innsending_id
and n.enkeltregning_id = e.enkeltregning_id
and i.fagomraade_kode IN ('PO','LR')
and n.undersokelseskode in ('SNG0AG')
and e.enkeltregning_status_k in ('godkjent', 'manuelt_godkjent')
and e.datotid >= '01.07.2021'
and e.datotid < '01.01.2022'
group by e.pasient_identifikasjon, i.fagomraade_kode
) r, odb_enkeltregning partition(y2021) e, odb_diagnose partition(y2021) d, odb_innsending i where 1=1
and e.innsending_id = i.innsending_id
and e.enkeltregning_id = d.enkeltregning_id
and r.pasient_identifikasjon = e.pasient_identifikasjon
and i.fagomraade_kode IN('LE','PO')
and e.datotid > r.forste_behandling
and e.datotid < add_months(r.forste_behandling,6)
and d.diagnose_kode like('M17%')
and not exists(select 0 from odb_ncrpkode partition(y2021) n2, odb_enkeltregning partition(y2021) e2 where 1=1
and n2.enkeltregning_id = e2.enkeltregning_id
and e2.pasient_identifikasjon = r.pasient_identifikasjon
and n2.undersokelseskode in ('SNG0AA')
and e2.datotid < r.forste_behandling
and e2.datotid > add_months(r.forste_behandling,-6)
)
group by r.pasient_identifikasjon;
```
