# Supplementary material for: Low-value MRI of the knee in Norway: a register-based study to identify the proportion of potentially low-value MRIs and estimate the related costs
Source: BMJ Open. 2024 Mar 13;14(3):e081860. doi: 10.1136/bmjopen-2023-081860 (PMC10941154; doi:10.1136/bmjopen-2023-081860)
Supplement: Author's manuscript [file bmjopen-2023-081860.draft_revisions.pdf]

BMJ Open is committed to open peer review. As part of this commitment we make the peer review history of every article we publish publicly available.

When an article is published we post the peer reviewers' comments and the authors' responses online. We also post the versions of the paper that were used during peer review. These are the versions that the peer review comments apply to.

The versions of the paper that follow are the versions that were submitted during the peer review process. They are not the versions of record or the final published versions. They should not be cited or distributed as the published version of this manuscript.

BMJ Open is an open access journal and the full, final, typeset and author-corrected version of record of the manuscript is available on our site with no access controls, subscription charges or pay-per-view fees (http://bmjopen.bmj.com).

If you have any questions on BMJ Open's open peer review process please email info.bmjopen@bmj.com

BMJ Open

Low value MRI of the knee: How much? A register-based study of the extension and costs of low value MRI of the knee in Norway

Journal:	*BMJ Open*
Manuscript ID	bmjopen-2023-081860
Article Type:	Original research
Date Submitted by the Author:	08-Nov-2023

Complete List of Authors:	Hofmann , Bjørn; Norwegian University of Science and Technology Faculty of Medicine and Health Sciences, Institute for the health sciences<br>Håvik, Vegard; Norwegian Directorate of Health, Department for Medical Reimbursement<br>Andersen, Eivind; Norwegian University of Science and Technology, Department of Health Sciences in Gjøvik<br>Brandsæter, Ingrid; Norwegian University of Science and Technology, Department of Health Sciences in Gjøvik<br>Kjelle, Elin; Norwegian University of Science and Technology, Department of Health Sciences in Gjøvik

Keywords:	Knee < ORTHOPAEDIC & TRAUMA SURGERY, Health Services, Health policy < HEALTH SERVICES ADMINISTRATION & MANAGEMENT, Quality in health care < HEALTH SERVICES ADMINISTRATION & MANAGEMENT, Magnetic resonance imaging < RADIOLOGY & IMAGING, RADIOLOGY & IMAGING

SCHOLARONE™
Manuscripts

I, the Submitting Author has the right to grant and does grant on behalf of all authors of the Work (as defined in the below author licence), an exclusive licence and/or a non-exclusive licence for contributions from authors who are: i) UK Crown employees; ii) where BMJ has agreed a CC-BY licence shall apply, and/or iii) in accordance with the terms applicable for US Federal Government officers or employees acting as part of their official duties; on a worldwide, perpetual, irrevocable, royalty-free basis to BMJ Publishing Group Ltd ("BMJ") its licensees and where the relevant Journal is co-owned by BMJ to the co-owners of the Journal, to publish the Work in this journal and any other BMJ products and to exploit all rights, as set out in our licence.

The Submitting Author accepts and understands that any supply made under these terms is made by BMJ to the Submitting Author unless you are acting as an employee on behalf of your employer or a postgraduate student of an affiliated institution which is paying any applicable article publishing charge ("APC") for Open Access articles. Where the Submitting Author wishes to make the Work available on an Open Access basis (and intends to pay the relevant APC), the terms of reuse of such Open Access shall be governed by a Creative Commons licence – details of these licences and which Creative Commons licence will apply to this Work are set out in our licence referred to above.

Other than as permitted in any relevant BMJ Author's Self Archiving Policies, I confirm this Work has not been accepted for publication elsewhere, is not being considered for publication elsewhere and does not duplicate material already published. I confirm all authors consent to publication of this Work and authorise the granting of this licence.

For peer review only

Low value MRI of the knee: How much?

A register-based study of the extension and costs of low value MRI of the knee in Norway

Short title:

Extension and costs of low value MRI of the knee in Norway

Authors:

Bjørn Hofmann[1,2]
Vegard Håvik[3]
Eivind Richter Andersen[2]
Ingrid Øfsti Brandsæter[2]
Elin Kjelle[2]

1. Centre of Medical Ethics at the University of Oslo, Norway, b.m.hofmann@medisin.uio.no
2. Institute for the Health Sciences at the Norwegian University of Science and Technology (NTNU) at Gjøvik, Norway, bjoern.hofmann@ntnu.no
3. Department for Medical Reimbursement, Norwegian Directorate of Health

Corresponding author:
Bjørn Hofmann,
Centre of Medical Ethics at the Faculty of Medicine
University of Oslo
PO Box 1130
N-0318 Oslo, Norway, b.m.hofmann@medisin.uio.no

For peer review only

Low-value MRI of the knee: How much?

A register-based study of the extension and costs of low-value MRI of the knee in Norway

Short title: Extension and costs of low value MRI of the knee in Norway

Abstract

Objectives: the objective of this study is to investigate the proportion MRI imaging of the knee that are of low value to patients in Norway and to provide an estimate of the related costs.

Design: Register study based on conditional data extraction and analysis of data from Control and Reimbursement of Healthcare Claims (CRHC) registry in Norway.

Setting: MRI imaging in public specialist healthcare with universal health coverage (Norway).

Participants: 48,212 MRIs for 41,456 unique patients and 45,946 reimbursement claims.

Primary and secondary outcome measures: proportion of MRIs of the knee that 1) did not have a relevant tentative diagnosis prior to the knee MRI, or 2) did not have a relevant alternative image of the knee before the MRI, or 3) did not have a relevant code from the specialist care within six months after the MRI, and those that had combinations of 1, 2, and 3. Estimated costs for those that had combinations of 1, 2, and 3.

Results: Between 58% and 85% of patients having a knee MRIs in Norway have low value imaging, as they have no relevant diagnoses or actions prior or after the MRI examination. This corresponds to between 24,108 and 35.416 MRIs at a cost of 6,7 million - 9,8 EUR per year. Very few patients (6.4%) had a relevant diagnosis code or prior imaging examination when having the MRI and only 14.6% got a knee-related diagnosis code from the specialist care within six months after the MRI.

Conclusion: A substantial part of MRIs of the knee in Norway is of low value. Reducing low-value MRIs could free resources for high-value imaging, reduce waiting times, improve the quality of care, and increase patient safety and professional integrity.

Strengths and limitations of this study

- Unique national estimates of the extension and costs of low-value MRI imaging of the knee
- Uniform coding practices
- Does not catch “do nothing” as an “intervention”
- Specific to public healthcare setting with universal coverage

Key words: MRI, knee, low value care, Choosing Wisely

For peer review only - http://bmjopen.bmj.com/site/about/guidelines.xhtml

Low-value MRI of the knee: How much?

A register-based study of the extension and costs of low-value MRI of the knee in Norway

Background

Radiological examinations are invaluable in modern patient care, as they facilitate accurate diagnostics, expedite crucial treatment, and reduce morbidity and mortality.[1] On the other hand, uncritical and inappropriate use of radiology may reduce quality and safety of care, e.g., in terms of false (positive and negative) results, misdiagnosis, incidental findings of uncertain significance, overdiagnosis, delays in appropriate care, and increased costs.[2 3] Accordingly, it is crucial to reduce the use of low-value examinations, i.e., examinations that do not lead to change in patient management or is deemed beneficial.[4]

Magnetic resonance imaging (MRI) of the knee for knee pain without red flags is recognized as low-value by the international Choosing Wisely campaign and is the second most commonly studied musculoskeletal low-value examination worldwide.[5] Eight studies reported a change in treatment in less than 1% of the cases after an MRI of the knee.[6-13] Further, imaging practice does not always concur with other health related practices. While there has been a reduction in knee arthroscopy, the number of knee MRIs has increased substantially, and a recent study from Australia reported a seven-fold increase from 2003-2017.[11] A large proportion of low-value knee MRIs is an indicator of poor-quality care, waste of resources, and a cause of unnecessary waiting times and delayed diagnosis and treatment. While MRI of the knee is an acknowledged low-value service, we need more knowledge on the extension and costs of this barrier to effective and efficient care.

This knowledge is crucial for targeting the problem of low-value imaging. Many studies document geographical variations in the use of knee MRIs,[14 15] and such variations indicate both underuse and overuse of services.[16 17] However, potential low-value imaging is not the same as actual low-value imaging as MRIs of the knee can be of high value to many patients. Hence, we need to investigate the actual low-value use of knee MRIs in context. Moreover, cost from unnecessary MRIs from single consultants in the NHS have been estimated,[18] but we need aggregated national estimates.

Therefore, it is crucial to document how many of the MRIs of the knee that are of low value and what the costs are. Accordingly, *the objective of this study is to investigate the proportion of low-value MRI imaging of the knee in Norway and to provide an estimate of the related costs*. The research questions are: 1) how large is the extension of low-value MRI imaging of the knee in Norway, and 2) what are the related costs?

Methods

Setting

In Norway, all residents are entitled to essential medical care,[19] and due to universal health coverage, most health services are publicly funded through taxes.[20] Specialists and radiological services are organized within the specialist care, governed by the regional hospital trusts.[21] Physiotherapist and general practitioners (GP) are organized in the primary care which are the

For peer review only - http://bmjopen.bmj.com/site/about/guidelines.xhtml

municipalities' responsibility. However, several orthopedist and physiotherapist clinics operate in the private health care sector either on contracts with the public health system or independently.[22]

Private imaging centers perform about 20-25% of all outpatient examinations, where 60% of these are Computed Tomography (CT) or MRI-examinations.[23] A significant portion of these examinations is commissioned by public health services to ease the pressure on public services.[21] Further, private imaging centers offer insurance or out-of-pocket financed radiology, which allows shorter waiting times for patients. About 10 percent of the population have private health insurances.[20]

Data

This study uses data from Control and Reimbursement of Healthcare Claims (CRHC) registry by the Norwegian Directorate of Health. Specified types of registry data were retrieved for all patients that performed an outpatient knee MRI at public hospitals and/or private imaging centers during a six-month period (July 1 to December 31, 2021). Only publicly employed healthcare providers or providers with contracts with the public health system report to CRHC. Independent orthopaedists or physiotherapists do not report to CRHC and are thus not included in this study.

Both hospitals and private imaging centers report radiological examinations to CRHC by using the Norwegian Classification of Radiological Procedures (NCRP)-codes. In this study the NCRP code for knee, MRI - SNG0AG, was used to extract relevant procedures.

Analysis

To estimate the proportion of knee MRIs that potentially are of low value, we identified all patients that 1) did not have a relevant tentative diagnosis prior to the knee MRI, or 2) did not have a relevant alternative image of the knee before the MRI, or 3) did not have a relevant code from the specialist care within six months after the MRI. We then investigated combinations of 1-3. The logic model of the data retrieval is illustrated in Figure 1.

[Please place Figure 1 here]

Figure 1 Illustration of the logic model of the data retrieval and indication of how two of the estimates of the proportion of low value imaging were calculated.

To identify the potentially valuable MRIs, we used diagnosis codes reported from GPs and physiotherapists (ICPC-2 codes: L78 and L96) within 6 months prior to the MRI and diagnosis codes reported from orthopaedic clinics recorded within 6 months after the MRI was performed (ICD-10 codes: M22, M23, M66.0, M70.5, M71.2 and M92.4). Moreover, we identified patients who had an x-ray, CT, or ultrasound of the knee within six months prior to the MRI, as we assumed these patients would have some value from the MRI. These examinations were identified through NCRP codes: SNG0AA, SNG0AG and SNG0AK.

Correspondingly, we analysed the number of patients that did neither have relevant imaging codes withing six months prior to the MRI nor have any of the relevant codes from the specialist care within six months after the MRI.

Additionally, we investigated the number of patients that did not have a relevant code from their GP or physiotherapist or relevant imaging codes within six months prior to the MRI and did not have a relevant code from the specialist care within six months after the MRI.

An overview of the radiological and diagnosis codes is given in Table 1.

For peer review only - http://bmjopen.bmj.com/site/about/guidelines.xhtml

Code	Description	Reason for subtracting patients with this code
Codes registered within 6 months prior to MRI		
NCRP - SNG0AA	X-ray of the knee	Findings or lack of findings on these examinations could indicate an MRI is appropriate
NCRP - SNG0AG	CT of the knee	
NCRP - SNG0AK	Ultrasound of the knee	
ICPC-2 L78	Distortion of the knee	MRI for these conditions could be considered appropriate
ICPC-2 L96	Acute inner knee damage	
Codes registered within 6 months after MRI		
ICD-10 M22	Disorders of patella	MRI for these conditions could be considered appropriate
ICD-10 M23	Internal derangement of knee	
ICD-10 M66.0	Rupture of popliteal cyst	
ICD-10 M70.5	Other bursitis of knee	
ICD-10 M71.2	Synovial cyst of popliteal space [Baker]	
ICD-10 S83.2	Tear of meniscus, current	
ICD-10 M92.4	Juvenile osteochondrosis of patella	

Table 1 Description of radiological and diagnosis codes and reason why these codes are used to identify appropriate MRI examinations. (MRI- Magnetic resonance imaging, CT-computed tomography)

Cost estimates were based on public cost per patient data for MRI in Norway.

Conditional data extraction and analysis is done with SQL-developer version 21.4.1.349.

Results

A total number of 48,212 MRIs were conducted during the period, divided by 41,456 unique patients and 45,946 reimbursement claims (used for categorizing reimbursements from CRHC).

A total of 33,234 (80.2%) patients did neither have relevant codes (L78, L96) from their GP or physiotherapist within a period of six months prior to the MRI nor have any of the relevant diagnosis codes from the specialist care within six months after the MRI.

A total of 28,140 (67.9%) patients did neither have relevant imaging codes (SNG0AA, SNG0AD, SNG0AK) within six months prior to the MRI nor have any of the relevant codes from the specialist care in Table 1 within six months after the MRI.

A total of 26,786 (64.6%) patients did not have a relevant code from their GP or physiotherapist or relevant imaging codes within six months prior to the MRI and did not have a relevant code from the specialist care within six months after the MRI.

Moreover, we found that 6,040 (14.6%) patients got a relevant diagnosis code for the knee within the first six months after the MRI. 2,201 (5.3%) patients had a relevant diagnosis code for the knee from their GP prior to the MRI, and 461 (1.1%) patients had relevant codes from a publicly registered physiotherapist. 9,016 (21.8%) patients had knee X-ray, CT, or ultrasound within six months before the MRI. Figure 2 provides an overview of these results.

[Please place Figure 2 here]

Figure 2 Illustration of the relationship between the various results

In 2021 the total number of outpatient MRIs of the knee were 87,140. Mean cost per patient for knee MRI in 2021 at public hospitals were 362 EUR (428 USD), and at private imaging centres 256

For peer review only - http://bmjopen.bmj.com/site/about/guidelines.xhtml

EUR (303 USD). In 2021, approximately 80% of outpatient MRIs if the knee were conducted at different private imaging centres. Based on this distribution, the total number of knee MRI amounts to a cost of 23,8 million EUR (28,2 million USD).

Interpretation

Depending on how you define low-value imaging related to these findings, you come out with different estimates of the proportion of low value MRIs.

If all cases of MRI for persons who have no healthcare-relevant implications of the imaging within six months after the MRI count as low value, 85.43% ((41456-6040)/41456) of all patients had a low-value MRI.

If patients that did neither have relevant imaging codes within six months prior to the MRI nor have any of the relevant codes from the specialist care within six months after the MRI count as low value, then 67.88% (28,140/41,456) of all patients had a low-value MRI.

Moreover, if those cases where patients that did not have a relevant code from their GP or physiotherapist or relevant imaging codes within six months prior to the MRI and did not have a relevant code from the specialist care within six months after the MRI count as low value, then 64.61% (26,786/41,456) of all patients had a low-value MRI.

Additionally, if the same percentage of those without any codes within six months before or after the MRI had private insurance as the general population in Norway (10%), and all of these did receive healthcare services from private providers not reporting to CRHC (n=2,678), and if none of these were of low value, then 58.15% of all patients had a low-value MRI.

Table 2 provides an overview of the various estimates of the proportion of low-value MRIs and the corresponding yearly costs.

Estimate	Very conservative	Conservative	Moderate	Liberal
Proportion (%)	58.15	64.61	67.88	85.43
Number of low value MRIs	24,108	26,786	28,140	35,416
Estimation	= (26786-2,678) / 41,456	= 26786/41456	= 28,140/41,456	= (41456-6040)/ 41456
Cost estimates in million EUR, (USD)	6,7 (7,9)	7,4 (8,8)	7,8 (9,2)	9,8 (11,6)

Table 2 Overview of the estimates of the proportion of low-value MRIs and the corresponding yearly costs

Discussion

This analysis of registry data showed that very few patients (6.4%) had a relevant diagnosis or imaging code registered prior to an outpatient MRI, and that 14.6% got a knee-related diagnosis code from the specialist care within six months after the MRI. Between 58% and 85% of the MRIs of the knee are estimated to be of low value at a cost of 6,7 – 9,8 million EUR (7,9 – 11,6 million USD).

For peer review only - http://bmjopen.bmj.com/site/about/guidelines.xhtml

The findings are in line with the international literature showing that 40-95% of knee MRIs were inappropriate or unnecessary.[6-13] The results (85% low value MRIs of the knee) are also in line with the results of an internal unpublished report at one of the major hospitals in Norway (2005).

In 2004, the rate of knee MRIs was 156,[24] while the average number increased to 173 for the period 2012-2015.[21] Interestingly, the overall annual rate of arthroscopic procedures declined by 33 % from 2012 to 2016.[25] Further, geographical variations in knee MRIs conducted in Norway are well documented, ranging from 117 to 211 examinations per 10 000 inhabitants.[21]

Accordingly, the findings fit with the documented discrepancy between imaging use and outcome.[5 26 27] Extensive use of imaging with low direct outcomes may indicate that patients and clinicians acquire imaging without a plan for utilizing the results on a nice-to-know basis.[28] Unfortunately, our findings do not falsify this hypothesis as only about 6% of the patients had a tentative diagnosis code for the knee from their GP or physiotherapist prior to the MRI examination.

Disturbingly, the results may also indicate a practice where doctors are afraid of giving a diagnosis based on clinical examinations without imaging, i.e., lowering clinical confidence and competency and weakening the gate-keeper function. According to the Norwegian guidelines for musculoskeletal imaging explicitly state that clinical assessment is the primary diagnostic tool.[29]

Interestingly, only about 22% of the patients had another type of knee imaging prior to the MRI. This finding indicates that advanced imaging (MRI) is referrers primary choice of imaging. However, for a common specific conditions, knee osteoarthritis, the primary imaging modality is x-ray, not MRI.[29] This adds to suggested practice change in the literature on low-value imaging, proposing taking x-ray before conducting knee MRI as a warranted way to optimize the use of imaging examinations.

Our very conservative estimate shows that more than 58% of MRI are potentially of low value, but the real number of low-value knee MRIs is likely higher. International studies shows that less than 1% of knee MRI results yielded change in patient management.[5] Thus, the potential for shfting resources from low-value MRIs to high-value MRIs in the Norwegian healthcare system in a safe manner is substantial.

Limitations

In this study, we have made a series of assumptions. Firstly, we assumed that patients followed up in the specialist healthcare system with relevant knee-related (ICD) codes within six months after the MRI would have some value from the examination. This assumption may not hold, as the diagnoses may have been set from other examinations than the MRI. If so, our estimates of low-value MRIs would be too low. On the other hand, diagnoses from the specialist healthcare may have been set more than six months after the MRI, resulting in overestimating low-value imaging. However, waiting times are much shorter than six months (especially in private institutions), so this may be unlikely.

Secondly, we assumed that an MRI would be indicated for patients with relevant diagnostic codes related to the knee from their GP or physiotherapist within six months prior to the MRI. This may not hold, as diagnoses may be set to provide reasons for having a MRI (e.g., if requested by the patient).[30]

Thirdly, we also assumed that patients who had an x-ray, CT, or ultrasound of their knee within six months prior to the MRI would have some value from the examination. This may not always be the case, making us underestimate the proportion of low-value imaging.

For peer review only - http://bmjopen.bmj.com/site/about/guidelines.xhtml

Fourthly, our data did not include data from independent orthopaedists and physiotherapists. This would lead to an over estimation of low value MRIs as some patients might be treated for relevant diagnosis in the private health services. To compensate for this, we assumed a 10% rate of patients treated in private independent healthcare and that all of these are of high value.

Fifthly, some examinations may have "do nothing" as an outcome, e.g., as an alternative to an intervention. This would mimic a low-value examination in our study. However, if "do nothing" follow a tentative diagnosis before the MRI, they do not count as low-value care, as the care pathway is changed.

Finally, data from in-patient examinations or examinations paid out-of-pocket were not included. The number of in-patient examinations of knee MRIs is very low, and the out-of-pocket examinations are not relevant in a study on the public healthcare system with a societal perspective.

Another source of error may be that there are other codes that are relevant than the ones we have identified, or that clinical conditions are under-reported. To reduce the first problem, we have discussed the codes with experts in the field. The second source of error may be small as there is a high coding consciousness in Norway as reimbursements depend on reporting of codes.[31] While the clinical information about patients is limited in this study, the model used for identifying appropriateness is assessed as relevant to overcome this challenge in identifying potential low-value knee MRIs. From related areas we know that coding may be inaccurate,[32] however, we have no knowledge about this source of poor performance.

The study is conducted just after most restrictions of the corona virus pandemic (2020-2021) was lifted and a backlog of MRIs could be expected. However, this should have resulted in a higher number of high-value MRIs of the knee. As seen from another study, high value imaging did not have specific priority under the pandemic.[33]

It is important to note that this study is performed in a public health care system with universal coverage. The premises, measures, and outcomes may be different from other settings.

Conclusion

Between 58% and 85% of patients having a knee MRIs in Norway have low value imaging, as they have no relevant diagnoses or actions prior or after the MRI examination. Very few patients (6.4%) had a relevant diagnosis code or prior imaging examination when having the MRI and only 14.6% got a knee-related diagnosis code from the specialist care within six months after the MRI.

The total number of low-value MRIs of the knee per year would be between 24,108 and 35.416, and amount to a cost of 6,7 million - 9,8 EUR for 2021.

A substantial part of MRIs of the knee in Norway is of low value. Reducing low-value MRIs could free resources for high-value imaging, reduce waiting times, improve the quality of care, and increase patient safety and professional integrity.

List of abbreviations

MRI: Magnetic Resonance Imaging
CT: Computed Tomography
GP: General Practitioners
CRHC: Control and Reimbursement of Healthcare Claims
NCRP: Norwegian Classification of Radiological Procedures

For peer review only - http://bmjopen.bmj.com/site/about/guidelines.xhtml

Declarations

Ethics approval: No patient data were handled outside the data system of the data owner, i.e., the Norwegian Directorate of Health. Accordingly, ethics approval is not applicable according to Norwegian law. Regional Ethics Committee (IRB) approval is regulated by the Norwegian Health Research Act. However, this study does not fall under this act as it does not "provide new knowledge about health and disease" (§4a) but is health services research.

Consent to participate: No patients are involved in this study. Individual patient data are not transferred outside the healthcare data registries. Analyses are performed by data owner within the data system of the Directorate of health. No patient data, and only aggregate (numerical count) data, are reported outside the system.

As no patients were involved (and no experimental protocols were used involving patients), the content of the research did not fall under the Norwegian Health Research Act, and no informed consent was required as no patient (data) was extracted to external researchers, no IRB (Regional Ethics Committee) approval was necessary for this study.

All methods were carried out in accordance with relevant guidelines and regulations.

Consent for publication: Not applicable

Availability of data and materials: All analyses results are available in the publication. The datasets in the health registries are available from CRHC on approved request. Please contact Vegard Håvik for guidance. The queries for data extraction and algorithms for data analyses are available on request to Vegard Håvik.

Competing interests: The authors declare that they have no conflicts of interest

Funding: This project received financial support from the Research Council of Norway (Project number 302503)

Patient and Public Involvement: Representatives of the Norwegian patient organization and an expert committee with representatives from the involved professions and authorities were involved on the research project from which this study stems.

Authors' contributions: BH designed the study with input from VH, ERA, IØB, and EK. VH performed the data analyses. BH wrote the first draft of the manuscript and all authors provided substantial input for several rounds of subsequent revisions.

Acknowledgement: We are most thankful to the Directorate of Health for their help with the analyses.

References

1. Brady A, Brink J, Slavotinek J. Radiology and Value-Based Health Care. *Jama* 2020;324(13):1286-87. doi: 10.1001/jama.2020.14930
2. Sajid IM, Parkunan A, Frost K. Unintended consequences: quantifying the benefits, iatrogenic harms and downstream cascade costs of musculoskeletal MRI in UK primary care. *BMJ open quality* 2021;10(3):e001287.
3. Hofmann B, Andersen ER, Kjelle E. Visualizing the Invisible: Invisible Waste in Diagnostic Imaging. *Healthcare* 2021;9(12):1693.
4. Colla CH. Swimming against the current--what might work to reduce low-value care? *The New England journal of medicine* 2014;371(14):1280-3. doi: 10.1056/NEJMp1404503 [published Online First: 2014/10/02]

For peer review only - http://bmjopen.bmj.com/site/about/guidelines.xhtml

5. Kjelle E, Andersen ER, Krokeide AM, et al. Characterizing and quantifying low-value diagnostic imaging internationally: a scoping review. *BMC Medical Imaging* 2022;22(1):73. doi: 10.1186/s12880-022-00798-2
6. Adelani MA, Mall NA, Brophy RH, et al. The Use of MRI in Evaluating Knee Pain in Patients Aged 40 Years and Older. *J Am Acad Orthop Surg* 2016;24(9):653-9. doi: 10.5435/JAAOS-D-15-00681 [published Online First: 2016/08/02]
7. Karel YH, Verkerk K, Endenburg S, et al. Effect of routine diagnostic imaging for patients with musculoskeletal disorders: A meta-analysis. *Eur J Intern Med* 2015;26(8):585-95. doi: 10.1016/j.ejim.2015.06.018 [published Online First: 2015/07/19]
8. Parent ME, Vezina F, Carrier N, et al. Indications for and clinical procedures resulting from magnetic resonance imaging of the knee in older patients: Are we choosing wisely? *Canadian Family Physician* 2018;64(3):e126-e32.
9. Refahi S, Kachooei AR, Farsadpour M, et al. Is prescription of knee MRI according to standard clinical guideline? *Acta Medica Mediterranea* 2016;32:1207-11.
10. Sheridan GA, Bisseru A, Glynn AA. The utility of MRI scans for a painful knee in the elderly patient. *Ir J Med Sci* 2020 doi: 10.1007/s11845-020-02261-z [published Online First: 2020/05/30]
11. Smith L, Barratt A, Buchbinder R, et al. Trends in knee magnetic resonance imaging, arthroscopies and joint replacements in older Australians: still too much low-value care? *ANZ J Surg* 2020;90(5):833-39. doi: 10.1111/ans.15712 [published Online First: 2020/02/18]
12. Solivetti FM, Guerrisi A, Salducca N, et al. Appropriateness of knee MRI prescriptions: clinical, economic and technical issues. *Radiol Med* 2016;121(4):315-22. doi: 10.1007/s11547-015-0606-1 [published Online First: 2015/12/15]
13. Gomez-Garcia JM, Gomez-Romero FJ, Arencibia-Jimenez M, et al. Appropriateness of magnetic resonance imaging requested by primary care physicians for patients with knee pain. *Int J Qual Health Care* 2018;30(7):565-70. doi: 10.1093/intqhc/mzy067 [published Online First: 2018/04/11]
14. O'Sullivan JW, Stevens S, Oke J, et al. Practice variation in the use of tests in UK primary care: a retrospective analysis of 16 million tests performed over 3.3 million patient years in 2015/16. *Bmc Medicine* 2018;16:1-9.
15. Gransjøen AM, Lysdahl KB, Hofmann BM. Geographical variations in the use of diagnostic imaging of musculoskeletal diseases in Norway. *Acta Radiologica* 2019;60(9):1153-58.
16. Hofmann BM, Gransjøen AM. Geographical variations in the use of outpatient diagnostic imaging in Norway 2019. *Acta Radiologica Open* 2022;11(2):20584601221074561. doi: 10.1177/20584601221074561
17. Hofmann BM. Ethical issues with geographical variations in the provision of health care services. *BMC medical ethics* 2022;23:127:1-10. doi: http://dx.doi.org10.1186/s12910-022-00869-7
18. Khan MM, Pincher B, Pacheco R. Unnecessary magnetic resonance imaging of the knee: How much is it really costing the NHS? *Annals of Medicine and Surgery* 2021;70:102736.
19. Sperre Saunes I, Karanikolos M, Sagan A. Norway: health system review. *Health Systems in Transition* 2020;22(1):1-163.
20. Tikkanen R, Osborn R, Mossialos E, et al. International profiles of health care systems. New York: The Commonwealth Fund 2020.
21. Office of the Auditor General of Norway. Office of the Auditor General of Norway investigation into the use of outpatient diagnostic imaging [Riksrevisjonens undersøkelse av bruken av poliklinisk bildediagnostikk] Oslo: Riksrevisjonen 2017.
22. Ringard Å, Sagan A, Sperre Saunes I, et al. Norway: health system review. 2013
23. Directorate of Health. Strategy for rational use of imaging services [Strategi for rasjonell bruk av bildediagnostikk]. Oslo: Directorate of Health 2019.
24. Espeland A, Natvig NL, Loge I, et al. Magnetic resonance imaging of the knee in Norway 2002-2004 (national survey): rapid increase, older patients, large geographic differences. *BMC*

For peer review only - http://bmjopen.bmj.com/site/about/guidelines.xhtml

health services research 2007;7:115. doi: 10.1186/1472-6963-7-115 [published Online First: 2007/07/31]
25. Holtedahl R, Brox JI, Aune AK, et al. Changes in the rate of publicly financed knee arthroscopies: an analysis of data from the Norwegian patient registry from 2012 to 2016. *BMJ open* 2018;8(6):e021199. doi: 10.1136/bmjopen-2017-021199
26. Levin DC, Rao VM. Reducing Inappropriate Use of Diagnostic Imaging Through the Choosing Wisely Initiative. *Journal of the American College of Radiology* 2017;14(9):1245-52. doi: 10.1016/j.jacr.2017.03.012
27. Rao VM, Levin DC. The Overuse of Diagnostic Imaging and the Choosing Wisely Initiative. *Annals of internal medicine* 2012;157(8):574-76.
28. Hendee WR, Becker GJ, Borgstede JP, et al. Addressing overutilization in medical imaging. *Radiology* 2010;257(1):240-5. doi: 10.1148/radiol.10100063 [published Online First: 2010/08/26]
29. Helsedirektoratet. Nasjonal faglig retningslinje for bildediagnostikk ved ikke-traumatiske muskel- og skjelettlidelser - Anbefalinger for primærhelsetjenesten. Helsedirektoratet.no: Helsedirektoratet, 2014.
30. Salerno S, Laghi A, Cantone M-C, et al. Overdiagnosis and overimaging: an ethical issue for radiological protection. *La radiologia medica* 2019;124(8):714-20.
31. Anthun KS, Bjørngaard JH, Magnussen J. Economic incentives and diagnostic coding in a public health care system. *International Journal of Health Economics and Management* 2017;17:83-101.
32. Roof MA, Lygrisse K, Keitel L, et al. How accurate is ICD-10 coding for revision total knee arthroplasty? *The Journal of Arthroplasty* 2021;36(12):3950-58.
33. Hofmann BM, Andersen ER, Kjelle E. What can we learn from the SARS-COV-2 pandemic about the value of specific radiological examinations? *BMC Health Services Research* 2021;21(1):10. doi: http://dx.doi.org10.1186/s12913-021-07190-w

For peer review only

For peer review only - http://bmjopen.bmj.com/site/about/guidelines.xhtml

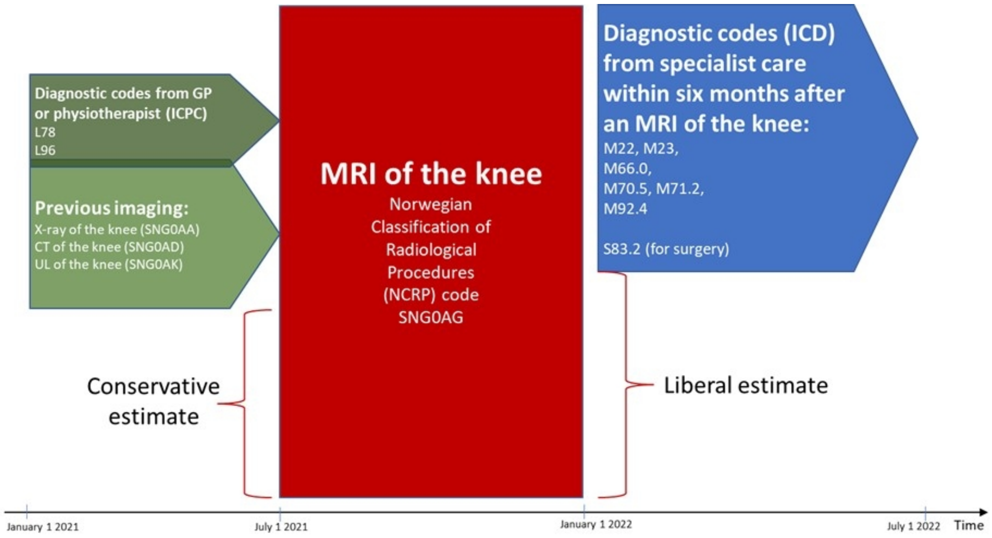

Figure 1 Illustration of the logic model of the data retrieval and indication of how two of the estimates of the proportion of low value imaging were calculated.

155x83mm (150 x 150 DPI)

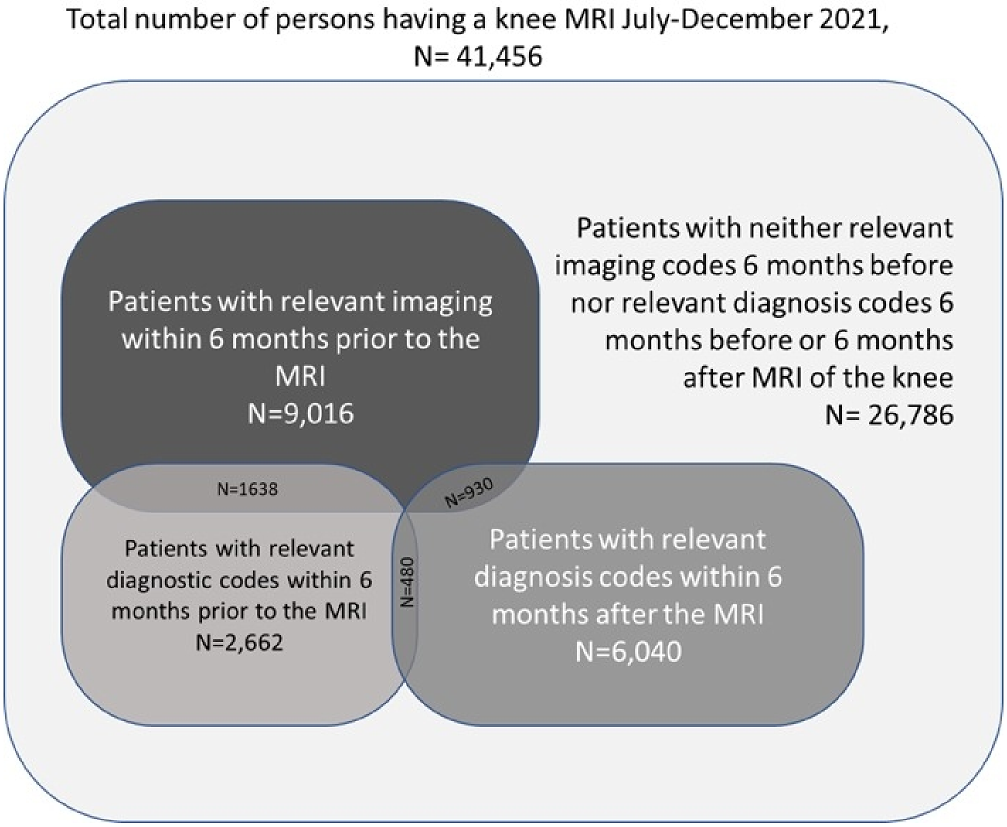

Figure 2 Illustration of the relationship between the various results

119x99mm (150 x 150 DPI)

Queries made by Department for Medical Reimbursement, Norwegian Directorate of Health

Lists pasientID and dates for MR Kne, point 1

```
select e.pasient_identifikasjon, to_char(e.datotid, 'YYYY-MM-DD') as dato
from odb_ncrpkode partition (y2021) n, odb_enkeltregning partition (y2021) e, odb_innsending i
where i.fagomraade_kode IN ('PO','LR') and
N.undersokelseskode in ('SNG0AG') and
i.innsending_status_k = 'godkjent' and
e.enkeltregning_status_k in ('godkjent', 'manuelt_godkjent') and
e.datotid >= '01.07.2021' and
e.datotid < '01.01.2022' and
n.enkeltregning_id = e.enkeltregning_id and
i.innsending_id = e.innsending_id
group by e.pasient_identifikasjon, to_char(e.datotid, 'YYYY-MM-DD')
order by 1,2;
```

Lists patients registered with selected diagnosis the last six moths after first MRI of the knee (SNG0AG), and removes the details to list relevant patients

```
select r.pasient_identifikasjon, r.forste_behandling, d.diagnose_kode, to_char(MIN(e.datotid),'dd.mm.yyyy') as
dato_diagnose from
(
select e.pasient_identifikasjon, MIN(e.datotid) as forste_behandling
from odb_ncrpkode partition (y2021) n, odb_enkeltregning partition (y2021) e, odb_innsending i
where 1=1
and i.innsending_id = e.innsending_id
and n.enkeltregning_id = e.enkeltregning_id
and i.fagomraade_kode IN ('PO','LR')
and n.undersokelseskode in ('SNG0AG')
and e.enkeltregning_status_k in ('godkjent', 'manuelt_godkjent')
and e.datotid >= '01.07.2021'
and e.datotid < '01.01.2022'
group by e.pasient_identifikasjon
) r, odb_enkeltregning e, odb_diagnose d, odb_innsending i where 1=1
and e.innsending_id = i.innsending_id
and e.enkeltregning_id = d.enkeltregning_id
and r.pasient_identifikasjon = e.pasient_identifikasjon
and i.fagomraade_kode IN('LE','PO')
and e.datotid > r.forste_behandling
and e.datotid < add_months(r.forste_behandling,6)
and d.diagnose_kode
IN('M220','M221','M222','M223','M224','M228','M229','M230','M231','M232','M233','M234','M235','M236','M238','M2
39','M660','M705','M712','M924','S832')
group by r.pasient_identifikasjon, r.forste_behandling, d.diagnose_kode;
```

Lists patients registered with specific diagnosis the first six months after the MRI of the knee – point 2 shows public/private distribution

```
select r.pasient_identifikasjon, r.fagomraade_kode from
(
select e.pasient_identifikasjon, i.fagomraade_kode, MIN(e.datotid) as forste_behandling
from odb_ncrpkode partition (y2021) n, odb_enkeltregning partition (y2021) e, odb_innsending i
where 1=1
and i.innsending_id = e.innsending_id
and n.enkeltregning_id = e.enkeltregning_id
and i.fagomraade_kode IN ('PO','LR')
and n.undersokelseskode in ('SNG0AG')
and e.enkeltregning_status_k in ('godkjent', 'manuelt_godkjent')
and e.datotid >= '01.07.2021'
and e.datotid < '01.01.2022'
group by e.pasient_identifikasjon, i.fagomraade_kode
) r, odb_enkeltregning e, odb_diagnose d, odb_innsending i where 1=1
and e.innsending_id = i.innsending_id
and e.enkeltregning_id = d.enkeltregning_id
and r.pasient_identifikasjon = e.pasient_identifikasjon
and i.fagomraade_kode IN('LE','PO')
and e.datotid > r.forste_behandling
and e.datotid < add_months(r.forste_behandling,6)
```

For peer review only

```
and d.diagnose_kode
IN('M220','M221','M222','M223','M224','M228','M229','M230','M231','M232','M233','M234','M235','M236','M238','M2
39','M660','M705','M712','M924','S832')
group by r.pasient_identifikasjon, r.fagomraade_kode;
```

Lists patients registered with specified diagnosis the six first months after MRI – point 3 shows shows those registered with a doctor/physiotherapist 6 months before the first SNG0AG, remove the details of the number of patients

```
select r.pasient_identifikasjon from
(
select e.pasient_identifikasjon, MIN(e.datotid) as forste_behandling
from odb_ncrpkode partition (y2021) n, odb_enkeltregning partition (y2021) e, odb_innsending i
where 1=1
and i.innsending_id = e.innsending_id
and n.enkeltregning_id = e.enkeltregning_id
and i.fagomraade_kode IN ('PO','LR')
and n.undersokelseskode in ('SNG0AG')
and e.enkeltregning_status_k in ('godkjent', 'manuelt_godkjent')
and e.datotid >= '01.07.2021'
and e.datotid < '01.01.2022'
group by e.pasient_identifikasjon
) r, odb_enkeltregning e, odb_diagnose d, odb_innsending i where 1=1
and e.innsending_id = i.innsending_id
and e.enkeltregning_id = d.enkeltregning_id
and r.pasient_identifikasjon = e.pasient_identifikasjon
and i.fagomraade_kode IN('LE')
and i.praksis_refusjonsgrunnlag in ('Fastlege','Fastlønnet','Turnuslege fastlønnet')
--and i.fagomraade_kode IN('LE','FY')
--and i.praksis_refusjonsgrunnlag not in ('Legevakt','Legevakt kommunal')
and e.datotid < r.forste_behandling
and e.datotid > add_months(r.forste_behandling,-6)
and d.diagnose_tabell in ('ICPC-2','ICPC-2B')
and (d.diagnose_kode like ('L78%') or
d.diagnose_kode like ('L96%'))
group by r.pasient_identifikasjon;group by r.pasient_identifikasjon, r.forste_behandling, d.diagnose_kode;
```

Lists the patients who are registered with SNG0AG, but who are not registered with any of the selected diagnostic codes six months before the first date with SNG0AG, point 3

```
select r.pasient_identifikasjon, r.forste_behandling from
(
select e.pasient_identifikasjon, MIN(e.datotid) as forste_behandling
from odb_ncrpkode partition (y2021) n, odb_enkeltregning partition (y2021) e
--, odb_innsending i
where 1=1
--and i.innsending_id = e.innsending_id
and n.enkeltregning_id = e.enkeltregning_id
--and i.fagomraade_kode IN ('PO','LR')
and e.enkeltregning_status_k in ('godkjent', 'manuelt_godkjent')
and n.undersokelseskode in ('SNG0AG')
and e.datotid >= '01.07.2021'
and e.datotid < '01.01.2022'
group by e.pasient_identifikasjon
) r where 1=1
and not exists(
select 0 from odb_enkeltregning partition (y2021) e, odb_diagnose partition (y2021) d, odb_innsending i where
1=1
and e.innsending_id = i.innsending_id
and e.enkeltregning_id = d.enkeltregning_id
and r.pasient_identifikasjon = e.pasient_identifikasjon
and i.fagomraade_kode IN('LE')
and i.praksis_refusjonsgrunnlag in ('Fastlege','Fastlønnet','Turnuslege fastlønnet')
--and i.fagomraade_kode IN('LE','FY')
--and i.praksis_refusjonsgrunnlag not in ('Legevakt','Legevakt kommunal')
and e.datotid < r.forste_behandling
and e.datotid > add_months(r.forste_behandling,-6)
and d.diagnose_tabell in ('ICPC-2','ICPC-2B')
and (d.diagnose_kode like ('L78%') or d.diagnose_kode like ('L96%'))
```

For peer review only

```
);
```

Lists patients registered with SNG0AG and registered with selected NCRP codes up to six months before the first date with SNG0AG, point 4

```
select r.pasient_identifikasjon from
(
select e.pasient_identifikasjon, MIN(e.datotid) as forste_behandling
from odb_ncrpkode partition (y2021) n, odb_enkeltregning partition (y2021) e, odb_innsending i
where 1=1
and i.innsending_id = e.innsending_id
and n.enkeltregning_id = e.enkeltregning_id
and i.fagomraade_kode IN ('PO','LR')
and n.undersokelseskode in ('SNG0AG')
and e.enkeltregning_status_k in ('godkjent', 'manuelt_godkjent')
and e.datotid >= '01.07.2021'
and e.datotid < '01.01.2022'
group by e.pasient_identifikasjon
) r, odb_enkeltregning e, odb_ncrpkode partition (y2021) n, odb_innsending i where 1=1
and e.innsending_id = i.innsending_id
and e.enkeltregning_id = n.enkeltregning_id
and r.pasient_identifikasjon = e.pasient_identifikasjon
and i.fagomraade_kode IN('PO','LR')
and e.datotid < r.forste_behandling
and e.datotid > add_months(r.forste_behandling,-6)
and n.undersokelseskode in ('SNG0AA','SNG0AD','SNG0AK')
group by r.pasient_identifikasjon;
```

How many in the sample above are not included in the UU1 sample, item 4

```
select count (distinct p.fnr)
from TMP_VH_23_HOFMANN_SML_MED_UU1 p where 1=1
and not exists(select 0 from TMP_VH_23_HOFMANN_UU1 r where 1=1
and p.fnr = r.fnr);
```

How many in the main sample have M17, but not SNG0AA

```
select r.pasient_identifikasjon from
(
select e.pasient_identifikasjon, i.fagomraade_kode, MIN(e.datotid) as forste_behandling
from odb_ncrpkode partition(y2021) n, odb_enkeltregning partition(y2021) e, odb_innsending i
where 1=1
and i.innsending_id = e.innsending_id
and n.enkeltregning_id = e.enkeltregning_id
and i.fagomraade_kode IN ('PO','LR')
and n.undersokelseskode in ('SNG0AG')
and e.enkeltregning_status_k in ('godkjent', 'manuelt_godkjent')
and e.datotid >= '01.07.2021'
and e.datotid < '01.01.2022'
group by e.pasient_identifikasjon, i.fagomraade_kode
) r, odb_enkeltregning partition(y2021) e, odb_diagnose partition(y2021) d, odb_innsending i where 1=1
and e.innsending_id = i.innsending_id
and e.enkeltregning_id = d.enkeltregning_id
and r.pasient_identifikasjon = e.pasient_identifikasjon
and i.fagomraade_kode IN('LE','PO')
and e.datotid > r.forste_behandling
and e.datotid < add_months(r.forste_behandling,6)
and d.diagnose_kode like('M17%')
and not exists(select 0 from odb_ncrpkode partition(y2021) n2, odb_enkeltregning partition(y2021) e2 where 1=1
and n2.enkeltregning_id = e2.enkeltregning_id
and e2.pasient_identifikasjon = r.pasient_identifikasjon
and n2.undersokelseskode in ('SNG0AA')
and e2.datotid < r.forste_behandling
and e2.datotid > add_months(r.forste_behandling,-6)
)
group by r.pasient_identifikasjon;
```

For peer review only

BMJ Open

Potentially low value MRI of the knee in Norway: a register-based study to identify the proportion of potentially low-value MRIs and estimate the related costs

Journal:	*BMJ Open*
Manuscript ID	bmjopen-2023-081860.R1
Article Type:	Original research
Date Submitted by the Author:	22-Feb-2024
Complete List of Authors:	Hofmann , Bjørn; Norwegian University of Science and Technology Faculty of Medicine and Health Sciences, Institute for the health sciences<br>Håvik, Vegard; Norwegian Directorate of Health, Department for Medical Reimbursement<br>Andersen, Eivind; Norwegian University of Science and Technology, Department of Health Sciences in Gjøvik<br>Brandsæter, Ingrid; Norwegian University of Science and Technology, Department of Health Sciences in Gjøvik<br>Kjelle, Elin; Norwegian University of Science and Technology, Department of Health Sciences in Gjøvik
**Primary Subject Heading**:	Health services research
Secondary Subject Heading:	Health policy, Health services research, Diagnostics, Medical management, Radiology and imaging
Keywords:	Knee < ORTHOPAEDIC & TRAUMA SURGERY, Health Services, Health policy < HEALTH SERVICES ADMINISTRATION & MANAGEMENT, Quality in health care < HEALTH SERVICES ADMINISTRATION & MANAGEMENT, Magnetic resonance imaging < RADIOLOGY & IMAGING, RADIOLOGY & IMAGING

SCHOLARONE™
Manuscripts

I, the Submitting Author has the right to grant and does grant on behalf of all authors of the Work (as defined in the below author licence), an exclusive licence and/or a non-exclusive licence for contributions from authors who are: i) UK Crown employees; ii) where BMJ has agreed a CC-BY licence shall apply, and/or iii) in accordance with the terms applicable for US Federal Government officers or employees acting as part of their official duties; on a worldwide, perpetual, irrevocable, royalty-free basis to BMJ Publishing Group Ltd ("BMJ") its licensees and where the relevant Journal is co-owned by BMJ to the co-owners of the Journal, to publish the Work in this journal and any other BMJ products and to exploit all rights, as set out in our licence.

The Submitting Author accepts and understands that any supply made under these terms is made by BMJ to the Submitting Author unless you are acting as an employee on behalf of your employer or a postgraduate student of an affiliated institution which is paying any applicable article publishing charge ("APC") for Open Access articles. Where the Submitting Author wishes to make the Work available on an Open Access basis (and intends to pay the relevant APC), the terms of reuse of such Open Access shall be governed by a Creative Commons licence – details of these licences and which Creative Commons licence will apply to this Work are set out in our licence referred to above.

Other than as permitted in any relevant BMJ Author's Self Archiving Policies, I confirm this Work has not been accepted for publication elsewhere, is not being considered for publication elsewhere and does not duplicate material already published. I confirm all authors consent to publication of this Work and authorise the granting of this licence.

For peer review only

Potentially low value MRI of the knee in Norway: a register-based study to identify the proportion of potentially low-value MRIs and estimate the related costs

Authors:

Bjørn Hofmann[1,2]
Vegard Håvik[3]
Eivind Richter Andersen[2]
Ingrid Øfsti Brandsæter[2]
Elin Kjelle[2]

1. Centre of Medical Ethics at the University of Oslo, Norway
2. Institute for the Health Sciences at the Norwegian University of Science and Technology (NTNU) at Gjøvik, Norway
3. Department for Medical Reimbursement, Norwegian Directorate of Health

Correspondence to:
Bjørn Hofmann,
Centre of Medical Ethics at the Faculty of Medicine
University of Oslo
PO Box 1130
N-0318 Oslo, Norway
b.m.hofmann@medisin.uio.no

For peer review only

Abstract

Objectives: The objective of this study is to investigate the proportion of potentially low-value knee MRI imaging in Norway and to provide an estimate of the related costs.

Design: Register study based on conditional data extraction and analysis of data from Control and Reimbursement of Healthcare Claims (CRHC) registry in Norway.

Setting: MRI imaging in public specialist healthcare with universal health coverage (Norway).

Participants: 48,212 MRIs for 41,456 unique patients and 45,946 reimbursement claims.

Outcome measures: Proportion of MRIs of the knee that 1) did not have a relevant tentative diagnosis prior to the knee MRI, or 2) did not have a relevant alternative image of the knee before the MRI, or 3) did not have a relevant code from the specialist care within six months after the MRI, and those that had combinations of 1, 2, and 3. Estimated costs for those that had combinations of 1, 2, and 3.

Results: Between 58% and 85% of patients having a knee MRIs in Norway have no relevant diagnoses or actions prior to or after the MRI examination. These examinations are unlikely to benefit patients and they correspond to between 24,108 and 35.416 MRIs at a cost of 6,7 million - 9,8 EUR per year. Very few patients (6.4%) had a relevant diagnosis code or prior imaging examination when having the MRI and only 14.6% got a knee-related diagnosis code from the specialist care within six months after the MRI.

Conclusion: A substantial proportion of MRIs of the knee in Norway have no relevant diagnoses or actions before or after the MRI and are potentially of low value. Reducing low-value MRIs could free resources for high-value imaging, reduce waiting times, improve the quality of care, and increase patient safety and professional integrity.

Strengths and limitations of this study

- This study is unique in providing estimates of the proportion and costs of low-value MRI imaging of the knee for a whole nation.
- High quality registers and uniform coding practices provide reliable overall estimates.
- The results are specific to a public healthcare setting with universal coverage.
- The study is based on aggregate data and not of detailed analyses of individual cases.
- Estimates are not true values as they are based on several specific assumptions.

Keywords: MRI, knee, low value care, Choosing Wisely

For peer review only - http://bmjopen.bmj.com/site/about/guidelines.xhtml

INTRODUCTION

Radiological examinations are invaluable in modern patient care, as they facilitate accurate diagnostics, expedite crucial treatment, and reduce morbidity and mortality.[1] On the other hand, uncritical and inappropriate use of radiology may reduce quality and safety of care, e.g., in terms of false (positive and negative) results, misdiagnosis, incidental findings of uncertain significance, overdiagnosis, delays in appropriate care, and increased costs.[2 3] Accordingly, it is crucial to reduce the use of low-value examinations, i.e., examinations that do not lead to change in patient management or are deemed not to be beneficial.[4]

Magnetic resonance imaging (MRI) of the knee for knee pain without red flags (e.g., without mechanical symptoms or effusion, unless the patient has not improved after completion of an rehabilitation program) is recognized as low-value by the international Choosing Wisely campaign[5 6] and is the second most commonly studied musculoskeletal low-value examination worldwide.[7] Eight studies reported a change in treatment in less than 1% of the cases after an MRI of the knee.[8-15] Further, imaging practice does not always concur with other health related practices. While there has been a reduction in knee arthroscopy for specific conditions and age groups, the number of knee MRIs has increased substantially, and a recent study from Australia reported a seven-fold increase from 2003-2017.[13] While some of these MRIs might have influenced patient management decisions, such as shifting from arthroscopy to arthroplasty, it remains questionable whether such a volume of MRIs significantly improves patient care.

Accordingly, a large proportion of low-value knee MRIs is an indicator of poor-quality care, waste of resources, and a cause of unnecessary waiting times and delayed diagnosis and treatment. We therefore need more knowledge about the proportion and costs of this barrier to effective and efficient care. This is crucial for identifying and targeting the problem and for directing health policy making (deimplementation, disinvestment).

Many studies document geographical variations in the use of knee MRIs.[16 17] While such variations indicate both underuse and overuse of services,[18 19] they do not indicate the “right level” or what is of low and high value. We therefore need more information on which examinations that are (not) valuable for patients, health providers, and the healthcare system. While detailed analyses of individual patients’ pathways can provide insights into the appropriateness of single examinations, this level of analysis is unfeasible on a national or aggregated level. Moreover, cost from unnecessary MRIs from single consultants in the NHS have been estimated,[20] but we need aggregated national estimates.

Consequently, there is a need to document both how many of the MRIs of the knee that potentially are of low value and what the costs are. Therefore, by using register data to identify patients that did not have relevant diagnoses or actions six months before or after an MRI of the knee, *the objective of this study is to estimate the proportion of examinations that are unlikely to have benefited patients and that potentially are of low value and to provide an estimate of the related costs*.

The research questions are: 1) how large is the proportion of the MRIs of the knee that do not have relevant diagnoses or actions six months before or after the examination in Norway, and 2) what are the related costs per year for these examinations?

For peer review only - http://bmjopen.bmj.com/site/about/guidelines.xhtml

METHODS

Setting

In Norway, all residents are entitled to essential medical care,[21] and due to universal health coverage, most health services are publicly funded through taxes.[22] Specialists and radiological services are organized within the specialist care, governed by the regional hospital trusts.[23] Physiotherapist and general practitioners (GP) are organized in the primary care which are the municipalities' responsibility. However, several orthopedist and physiotherapist clinics operate in the private health care sector either on contracts with the public health system or independently.[24]

Private imaging centers perform about 20-25% of all outpatient examinations, where 60% of these are Computed Tomography (CT) or MRI-examinations.[25] A significant portion of these examinations is commissioned by public health services to ease the pressure on public services.[23] Further, private imaging centers offer insurance or out-of-pocket financed radiology, which allows shorter waiting times for patients. About 10 percent of the population have private health insurances.[22]

Data

This study uses data from Control and Reimbursement of Healthcare Claims (CRHC) registry by the Norwegian Directorate of Health. Specified types of registry data were retrieved for all patients that performed an outpatient knee MRI at public hospitals and/or private imaging centers during a six-month period (July 1 to December 31, 2021). Only publicly employed healthcare providers or providers with contracts with the public health system report to CRHC. Independent orthopaedists or physiotherapists do not report to CRHC and are thus not included in this study.

Both hospitals and private imaging centers report radiological examinations to CRHC by using the Norwegian Classification of Radiological Procedures (NCRP)-codes. In this study the NCRP code for knee, MRI - SNG0AG, was used to extract relevant procedures.

Analysis

As it is not feasible on a national level to analyse the medical history, indications, and clinical pathway of all patients, we assume that the MRIs of the knee that do not have relevant diagnoses or actions six months before or after the examination are unlikely to have benefited the patient and are potentially low-value examinations.

Moreover, the analyses are based on the assumptions that 1) the MRI of the knee is appropriate and of high value if the patient has received a relevant diagnostic code before the MRI, 2) the MRI has been of value to the patient if the patient has a knee-related diagnostic code after the examination, 3) patients undergoing other imaging examinations prior to the MRI experience value from the additional MRI, 4) 10% of patients migrate to private treatment of the knee and are not identified in the public health registries, 5) imaging in patients with a tentative knee diagnosis that do not lead to any active interventions (do nothing), are considered to be of high value the pathways of the patients have changed.

Consequently, to estimate the proportion of knee MRIs that potentially are of low value, we identified all patients that 1) did not have a relevant tentative diagnosis prior to the knee MRI, or 2) did not have a relevant alternative image of the knee before the MRI, or 3) did not have a relevant

For peer review only - http://bmjopen.bmj.com/site/about/guidelines.xhtml

code from the specialist care within six months after the MRI. We then investigated combinations of 1-3. The logic model of the data retrieval is illustrated in Figure 1.

To identify the potentially valuable MRIs, we used diagnosis codes reported from GPs and physiotherapists (ICPC-2 codes: L78 and L96) within 6 months prior to the MRI and diagnosis codes reported from orthopaedic clinics recorded within 6 months after the MRI was performed (ICD-10 codes: M22, M23, M66.0, M70.5, M71.2 and M92.4). Moreover, we identified patients who had an x-ray, CT, or ultrasound of the knee within six months prior to the MRI, as we assumed these patients would have some value from the MRI, for example when referred to specialist care. These examinations were identified through NCRP codes: SNG0AA, SNG0AG and SNG0AK.

Correspondingly, we analysed the number of patients that did neither have relevant imaging codes withing six months prior to the MRI nor have any of the relevant codes from the specialist care within six months after the MRI and therefore excluded MRIs which could be deemed necessary by specialists.

Additionally, we investigated the number of patients that did not have a relevant code from their GP or physiotherapist or relevant imaging codes within six months prior to the MRI and did not have a relevant code from the specialist care within six months after the MRI.

An overview of the radiological and diagnosis codes is given in Table 1.

Code	Description	Reason for subtracting patients with this code
Codes registered within 6 months prior to MRI		
NCRP - SNG0AA	X-ray of the knee	Findings or lack of findings on these examinations could indicate an MRI is appropriate
NCRP - SNG0AG	CT of the knee	
NCRP - SNG0AK	Ultrasound of the knee	
ICPC-2 L78	Distortion of the knee	MRI for these conditions could be considered appropriate
ICPC-2 L96	Acute inner knee damage	
Codes registered within 6 months after MRI		
ICD-10 M22	Disorders of patella	MRI for these conditions could be considered appropriate
ICD-10 M23	Internal derangement of knee	
ICD-10 M66.0	Rupture of popliteal cyst	
ICD-10 M70.5	Other bursitis of knee	
ICD-10 M71.2	Synovial cyst of popliteal space [Baker]	
ICD-10 S83.2	Tear of meniscus, current	
ICD-10 M92.4	Juvenile osteochondrosis of patella	

Table 1 Description of radiological and diagnosis codes and reason why these codes are used to identify appropriate MRI examinations. (MRI- Magnetic resonance imaging, CT-computed tomography)

Cost estimates were based on public cost per patient data for MRI in Norway.

Conditional data extraction and analysis is done with SQL-developer version 21.4.1.349. The queries made by Department for Medical Reimbursement, Norwegian Directorate of Health, are provided in the supplemental file.

For peer review only

For peer review only - http://bmjopen.bmj.com/site/about/guidelines.xhtml

Patient and public involvement

Representatives of the Norwegian patient organization and an expert committee with representatives from the involved professions and authorities were involved on the research project from which this study stems.

RESULTS

A total number of 48,212 MRIs were conducted during the period, divided by 41,456 unique patients and 45,946 reimbursement claims (used for categorizing reimbursements from CRHC).

A total of 33,234 (80.2%) patients did neither have relevant codes (L78, L96) from their GP or physiotherapist within a period of six months prior to the MRI nor have any of the relevant diagnosis codes from the specialist care within six months after the MRI.

A total of 28,140 (67.9%) patients did neither have relevant imaging codes (SNG0AA, SNG0AD, SNG0AK) within six months prior to the MRI nor have any of the relevant codes from the specialist care in Table 1 within six months after the MRI.

A total of 26,786 (64.6%) patients did not have a relevant code from their GP or physiotherapist or relevant imaging codes within six months prior to the MRI and did not have a relevant code from the specialist care within six months after the MRI.

Moreover, we found that 6,040 (14.6%) patients got a relevant diagnosis code for the knee within the first six months after the MRI. 2,201 (5.3%) patients had a relevant diagnosis code for the knee from their GP prior to the MRI, and 461 (1.1%) patients had relevant codes from a publicly registered physiotherapist. 9,016 (21.8%) patients had knee X-ray, CT, or ultrasound within six months before the MRI. Figure 2 provides an overview of these results.

In 2021 the total number of outpatient MRIs of the knee were 87,140. Mean cost per patient for knee MRI in 2021 at public hospitals were 362 EUR (428 USD), and at private imaging centres 256 EUR (303 USD). In 2021, approximately 80% of outpatient MRIs if the knee were conducted at different private imaging centres. Based on this distribution, the total number of knee MRI amounts to a cost of 23,8 million EUR (28,2 million USD).

Interpretation

The MRIs of the knee that do not have relevant diagnoses or actions before or after the MRI and that these are not likely to be important to patients' health and are potentially of low value. However, the estimates of the proportion of low-value imaging depend on how you define low-value imaging.

If all cases of MRI for persons who have no healthcare-relevant implications of the imaging within six months after the MRI count as low value, 85.43% ((41456-6040)/41456) of all patients had a low-value MRI.

If patients that did neither have relevant imaging codes within six months prior to the MRI nor have any of the relevant codes from the specialist care within six months after the MRI count as low value, then 67.88% (28,140/41,456) of all patients had a low-value MRI.

Moreover, if those cases where patients that did not have a relevant code from their GP or physiotherapist or relevant imaging codes within six months prior to the MRI and did not have a

For peer review only - http://bmjopen.bmj.com/site/about/guidelines.xhtml

relevant code from the specialist care within six months after the MRI count as low value, then 64.61% (26,786/41,456) of all patients had a low-value MRI.

Additionally, if the same percentage of those without any codes within six months before or after the MRI had private insurance as the general population in Norway (10%), and all of these did receive healthcare services from private providers not reporting to CRHC (n=2,678), and if none of these were of low value, then 58.15% of all patients had a low-value MRI.

Table 2 provides an overview of the various estimates of the proportion of low-value MRIs and the corresponding yearly costs.

Estimate	Very conservative	Conservative	Moderate	Liberal
Proportion (%)	58.15	64.61	67.88	85.43
Number of low value MRIs	24,108	26,786	28,140	35,416
Estimation	= (26786-2,678) / 41,456	= 26786/41456	= 28,140/41,456	= (41456-6040)/ 41456
Cost estimates in million EUR, (USD)	6,7 (7,9)	7,4 (8,8)	7,8 (9,2)	9,8 (11,6)

Table 2 Overview of the estimates of the proportion of low-value MRIs and the corresponding yearly costs

DISCUSSION

This analysis of registry data showed that very few patients (6.4%) had a relevant diagnosis or imaging code registered prior to an outpatient MRI, and that 14.6% got a knee-related diagnosis code from the specialist care within six months after the MRI. Between 58% and 85% of the MRIs of the knee are estimated to be of low value at a cost of 6,7 – 9,8 million EUR (7,9 – 11,6 million USD).

The findings are in line with the international literature showing that 40-95% of knee MRIs were inappropriate or unnecessary.[8-15] The results (85% low value MRIs of the knee) are also in line with the results of an internal unpublished report at one of the major hospitals in Norway (2005).

In 2004, the rate of knee MRIs was 156,[26] while the average number increased to 173 for the period 2012-2015.[23] Interestingly, the overall annual rate of arthroscopic procedures declined by 33 % from 2012 to 2016.[27] Further, geographical variations in knee MRIs conducted in Norway are well documented, ranging from 117 to 211 examinations per 10 000 inhabitants.[23]

Accordingly, the findings fit with the documented discrepancy between imaging use and outcome.[5-7] Extensive use of imaging with low direct outcomes may indicate that patients and clinicians acquire imaging without a plan for utilizing the results on a nice-to-know basis.[28] Unfortunately, our findings do not falsify this hypothesis as only about 6% of the patients had a tentative diagnosis code for the knee from their GP or physiotherapist prior to the MRI examination.

Disturbingly, the results may also indicate a practice where doctors are afraid of giving a diagnosis based on clinical examinations without imaging, i.e., lowering clinical confidence and competency and weakening the gate-keeper function. According to the Norwegian guidelines for musculoskeletal imaging explicitly state that clinical assessment is the primary diagnostic tool.[29]

For peer review only - http://bmjopen.bmj.com/site/about/guidelines.xhtml

Interestingly, only about 22% of the patients had another type of knee imaging prior to the MRI. This finding indicates that advanced imaging (MRI) is referrers primary choice of imaging. However, for a common specific conditions, knee osteoarthritis, the primary imaging modality is x-ray, not MRI.[29] This adds to suggested practice change in the literature on low-value imaging, proposing taking x-ray before conducting knee MRI as a warranted way to optimize the use of imaging examinations.

Our very conservative estimate shows that more than 58% of MRI are potentially of low value, but the real number of low-value knee MRIs is likely higher. International studies shows that less than 1% of knee MRI results yielded change in patient management.[7] Thus, the potential for shifting resources from low-value MRIs to high-value MRIs in the Norwegian healthcare system in a safe manner is substantial.

Limitations

In this study, we have made a series of assumptions. Firstly, we assumed that patients followed up in the specialist healthcare system with relevant knee-related (ICD) codes within six months after the MRI would have some value from the examination. This assumption may not hold, as the diagnoses may have been set from other examinations than the MRI (or the patient may have had a knee accident during this period). If so, our estimates of low-value MRIs would be too low. On the other hand, diagnoses from the specialist healthcare may have been set more than six months after the MRI, resulting in overestimating low-value imaging. However, waiting times are much shorter than six months (especially in private institutions), so this may be unlikely.

Secondly, we assumed that an MRI would be indicated for patients with relevant diagnostic codes related to the knee from their GP or physiotherapist within six months prior to the MRI. This may not hold, as diagnoses may be set to provide reasons for having a MRI (e.g., if requested by the patient).[30]

Thirdly, we also assumed that patients who had an x-ray, CT, or ultrasound of their knee within six months prior to the MRI would have some value from the examination. This may not always be the case, making us underestimate the proportion of low-value imaging.

Fourthly, our data did not include data from independent orthopaedists and physiotherapists. This would lead to an over estimation of low value MRIs as some patients might be treated for relevant diagnosis in the private health services. To compensate for this, we assumed a 10% rate of patients treated in private independent healthcare and that all of these are of high value. However, many of these enter back into the public healthcare (with new MRIs) after private treatment and are included in our estimates. Hence, the very conservative estimate may provide too low estimates.

Fifthly, some examinations may have “do nothing” as an outcome, e.g., as an alternative to an active intervention. This would mimic a low-value examination in our study. However, if “do nothing” follow a tentative diagnosis before the MRI, they do not count as low-value care, as the care pathway is changed.

Finally, data from in-patient examinations or examinations paid out-of-pocket were not included. The number of in-patient examinations of knee MRIs is very low, and the out-of-pocket examinations are not relevant in a study on the public healthcare system with a societal perspective.

Another source of error may be that there are other codes that are relevant than the ones we have identified, or that clinical conditions are under-reported. To reduce the first problem, we have discussed the codes with experts in the field, and the codes have been suggested by specialists. The second source of error may be small as there is a high coding consciousness in Norway as

For peer review only - http://bmjopen.bmj.com/site/about/guidelines.xhtml

reimbursements depend on reporting of codes.[31]
While the clinical information about patients is limited in this study, the model used for identifying appropriateness is assessed as relevant to overcome this challenge in identifying potential low-value knee MRIs. From related areas we know that coding may be inaccurate,[32] however, we have no knowledge about this source of poor performance.

The study is conducted just after most restrictions of the corona virus pandemic (2020-2021) was lifted and a backlog of MRIs could be expected. However, this should have resulted in a higher number of high-value MRIs of the knee. As seen from another study, high value imaging did not have specific priority under the pandemic.[33]

It is important to note that this study is performed in a public health care system with universal coverage. The premises, measures, and outcomes may be different from other settings.

CONCLUSION

Between 58% and 85% of patients having a knee MRIs in Norway have potentially low value imaging, as they have no relevant diagnoses or actions prior or after the MRI examination. Very few patients (6.4%) had a relevant diagnosis code or prior imaging examination when having the MRI and only 14.6% got a knee-related diagnosis code from the specialist care within six months after the MRI.

The total number of low-value MRIs of the knee per year would be between 24,108 and 35.416, and amount to a cost of 6,7 million - 9,8 EUR for 2021.

A substantial part of MRIs of the knee in Norway is potentially of low value. Reducing low-value MRIs could free resources for high-value imaging, reduce waiting times, improve the quality of care, and increase patient safety and professional integrity.

List of abbreviations

MRI: Magnetic Resonance Imaging
CT: Computed Tomography
GP: General Practitioners
CRHC: Control and Reimbursement of Healthcare Claims
NCRP: Norwegian Classification of Radiological Procedures

Declarations

Ethics approval and consent to participate: No patient data were handled outside the data system of the data owner, i.e., the Norwegian Directorate of Health. Accordingly, ethics approval is not applicable according to Norwegian law. Regional Ethics Committee (IRB) approval is regulated by the Norwegian Health Research Act. However, this study does not fall under this act as it does not "provide new knowledge about health and disease" (§4a) but is health services research.

No patients are involved in this study. Individual patient data are not transferred outside the healthcare data registries. Analyses are performed by data owner within the data system of the Directorate of health. No patient data, and only aggregate (numerical count) data, are reported outside the system.

As no patients were involved (and no experimental protocols were used involving patients), the content of the research did not fall under the Norwegian Health Research Act, and no informed consent was required as no patient (data) was extracted to external researchers, no IRB (Regional Ethics Committee) approval was necessary for this study.

For peer review only - http://bmjopen.bmj.com/site/about/guidelines.xhtml

For peer review only

All methods were carried out in accordance with relevant guidelines and regulations.

Consent for publication: Not applicable.

Data availability statement: No additional data available. The queries for data extraction and algorithms for data analyses are available in in the supplemental file.

Competing interests: The authors declare that they have no competing interests.

Funding: This project received financial support from the Research Council of Norway (Project number 302503).

Contributors: BH designed the study with input from VH, ERA, IØB, and EK. VH performed the data analyses. BH wrote the first draft of the manuscript and all authors provided substantial input for several rounds of subsequent revisions.

Acknowledgements: We are most thankful to the Directorate of Health for their help with the analyses.

References

1. Brady A, Brink J, Slavotinek J. Radiology and Value-Based Health Care. Jama 2020;**324**(13):1286-87 doi: 10.1001/jama.2020.14930[published Online First: Epub Date]|.
2. Sajid IM, Parkunan A, Frost K. Unintended consequences: quantifying the benefits, iatrogenic harms and downstream cascade costs of musculoskeletal MRI in UK primary care. BMJ open quality 2021;**10**(3):e001287
3. Hofmann B, Andersen ER, Kjelle E. Visualizing the Invisible: Invisible Waste in Diagnostic Imaging. Healthcare 2021;**9**(12):1693
4. Colla CH. Swimming against the current--what might work to reduce low-value care? The New England journal of medicine 2014;**371**(14):1280-3 doi: 10.1056/NEJMp1404503[published Online First: Epub Date]|.
5. Levin DC, Rao VM. Reducing Inappropriate Use of Diagnostic Imaging Through the Choosing Wisely Initiative. Journal of the American College of Radiology 2017;**14**(9):1245-52 doi: 10.1016/j.jacr.2017.03.012[published Online First: Epub Date]|.
6. Rao VM, Levin DC. The Overuse of Diagnostic Imaging and the Choosing Wisely Initiative. Annals of internal medicine 2012;**157**(8):574-76
7. Kjelle E, Andersen ER, Krokeide AM, et al. Characterizing and quantifying low-value diagnostic imaging internationally: a scoping review. BMC Medical Imaging 2022;**22**(1):73 doi: 10.1186/s12880-022-00798-2[published Online First: Epub Date]|.
8. Adelani MA, Mall NA, Brophy RH, Halstead ME, Smith MV, Wright RW. The Use of MRI in Evaluating Knee Pain in Patients Aged 40 Years and Older. J Am Acad Orthop Surg 2016;**24**(9):653-9 doi: 10.5435/JAAOS-D-15-00681[published Online First: Epub Date]|.
9. Karel YH, Verkerk K, Endenburg S, Metselaar S, Verhagen AP. Effect of routine diagnostic imaging for patients with musculoskeletal disorders: A meta-analysis. Eur J Intern Med 2015;**26**(8):585-95 doi: 10.1016/j.ejim.2015.06.018[published Online First: Epub Date]|.
10. Parent ME, Vezina F, Carrier N, Masetto A. Indications for and clinical procedures resulting from magnetic resonance imaging of the knee in older patients: Are we choosing wisely? Canadian Family Physician 2018;**64**(3):e126-e32
11. Refahi S, Kachooei AR, Farsadpour M, et al. Is prescription of knee MRI according to standard clinical guideline? Acta Medica Mediterranea 2016;**32**:1207-11
12. Sheridan GA, Bisseru A, Glynn AA. The utility of MRI scans for a painful knee in the elderly patient. Ir J Med Sci 2020 doi: 10.1007/s11845-020-02261-z[published Online First: Epub Date]|.

For peer review only - http://bmjopen.bmj.com/site/about/guidelines.xhtml

13. Smith L, Barratt A, Buchbinder R, Harris IA, Doust J, Bell K. Trends in knee magnetic resonance imaging, arthroscopies and joint replacements in older Australians: still too much low-value care? ANZ J Surg 2020;**90**(5):833-39 doi: 10.1111/ans.15712[published Online First: Epub Date]|.
14. Solivetti FM, Guerrisi A, Salducca N, et al. Appropriateness of knee MRI prescriptions: clinical, economic and technical issues. Radiol Med 2016;**121**(4):315-22 doi: 10.1007/s11547-015-0606-1[published Online First: Epub Date]|.
15. Gomez-Garcia JM, Gomez-Romero FJ, Arencibia-Jimenez M, Navarro-Gracia JF, Sanchez-Molla M. Appropriateness of magnetic resonance imaging requested by primary care physicians for patients with knee pain. Int J Qual Health Care 2018;**30**(7):565-70 doi: 10.1093/intqhc/mzy067[published Online First: Epub Date]|.
16. O'Sullivan JW, Stevens S, Oke J, et al. Practice variation in the use of tests in UK primary care: a retrospective analysis of 16 million tests performed over 3.3 million patient years in 2015/16. Bmc Medicine 2018;**16**:1-9
17. Gransjøen AM, Lysdahl KB, Hofmann BM. Geographical variations in the use of diagnostic imaging of musculoskeletal diseases in Norway. Acta Radiologica 2019;**60**(9):1153-58
18. Hofmann BM, Gransjøen AM. Geographical variations in the use of outpatient diagnostic imaging in Norway 2019. Acta Radiologica Open 2022;**11**(2):20584601221074561 doi: 10.1177/20584601221074561[published Online First: Epub Date]|.
19. Hofmann BM. Ethical issues with geographical variations in the provision of health care services. BMC medical ethics 2022;**23:127**:1-10 doi: http://dx.doi.org10.1186/s12910-022-00869-7[published Online First: Epub Date]|.
20. Khan MM, Pincher B, Pacheco R. Unnecessary magnetic resonance imaging of the knee: How much is it really costing the NHS? Annals of Medicine and Surgery 2021;**70**:102736
21. Sperre Saunes I, Karanikolos M, Sagan A. Norway: health system review. Health Systems in Transition 2020;**22**(1):1-163
22. Tikkanen R, Osborn R, Mossialos E, Djordjevic A, Wharton G. *International profiles of health care systems*. New York: The Commonwealth Fund, 2020.
23. Office of the Auditor General of Norway. *Office of the Auditor General of Norway investigation into the use of outpatient diagnostic imaging [Riksrevisjonens undersøkelse av bruken av poliklinisk bildediagnostikk]* Oslo: Riksrevisjonen, 2017.
24. Ringard Å, Sagan A, Sperre Saunes I, Lindahl AK, Organization WH. Norway: health system review. 2013
25. Directorate of Health. *Strategy for rational use of imaging services [Strategi for rasjonell bruk av bildediagnostikk]*. Oslo: Directorate of Health, 2019.
26. Espeland A, Natvig NL, Loge I, Engebretsen L, Ellingsen J. Magnetic resonance imaging of the knee in Norway 2002-2004 (national survey): rapid increase, older patients, large geographic differences. BMC Health Serv Res 2007;**7**:115 doi: 10.1186/1472-6963-7-115[published Online First: Epub Date]|.
27. Holtedahl R, Brox JI, Aune AK, Nguyen D, Risberg MA, Tjomsland O. Changes in the rate of publicly financed knee arthroscopies: an analysis of data from the Norwegian patient registry from 2012 to 2016. BMJ open 2018;**8**(6):e021199 doi: 10.1136/bmjopen-2017-021199[published Online First: Epub Date]|.
28. Hendee WR, Becker GJ, Borgstede JP, et al. Addressing overutilization in medical imaging. Radiology 2010;**257**(1):240-5 doi: 10.1148/radiol.10100063[published Online First: Epub Date]|.
29. Helsedirektoratet. Nasjonal faglig retningslinje for bildediagnostikk ved ikke-traumatiske muskel- og skjelettlidelser - Anbefalinger for primærhelsetjenesten. Helsedirektoratet.no: Helsedirektoratet, 2014.
30. Salerno S, Laghi A, Cantone M-C, Sartori P, Pinto A, Frija G. Overdiagnosis and overimaging: an ethical issue for radiological protection. La radiologia medica 2019;**124**(8):714-20

For peer review only - http://bmjopen.bmj.com/site/about/guidelines.xhtml

31. Anthun KS, Bjørngaard JH, Magnussen J. Economic incentives and diagnostic coding in a public health care system. International Journal of Health Economics and Management 2017;**17**:83-101
32. Roof MA, Lygrisse K, Keitel L, et al. How accurate is ICD-10 coding for revision total knee arthroplasty? The Journal of Arthroplasty 2021;**36**(12):3950-58
33. Hofmann BM, Andersen ER, Kjelle E. What can we learn from the SARS-COV-2 pandemic about the value of specific radiological examinations? BMC Health Services Research 2021;**21**(1):10 doi: http://dx.doi.org10.1186/s12913-021-07190-w[published Online First: Epub Date]|.

Figure captions/legends

Figure 1 Illustration of the logic model of the data retrieval and indication of how two of the estimates of the proportion of low value imaging were calculated. Diagnostic codes from ICPC-2 and other examinations six months prior to the MRI of the knee make the examination count as high value. The same goes for listed ICD-codes within six months after the examination. The liberal estimate is based on the proportion of MRIs without healthcare-relevant implications of the imaging within six months after the MRI. The conservative estimate is based on the proportion of MRIs where patients did neither have a relevant code from their GP or physiotherapist or relevant imaging codes within six months prior to the MRI and nor a relevant code from the specialist care within six months after the MRI.

Figure 2 Illustration of the relationship between the various identified groups: patients with relevant diagnostic codes six months prior to MRI of the knee; patients with other relevant imaging six months prior to MRI of the knee; patients with relevant diagnostic codes within six months after the MRI of the knee; patients with neither of the above-mentioned qualifications. Overlaps are shown in number of images.

For peer review only

For peer review only - http://bmjopen.bmj.com/site/about/guidelines.xhtml

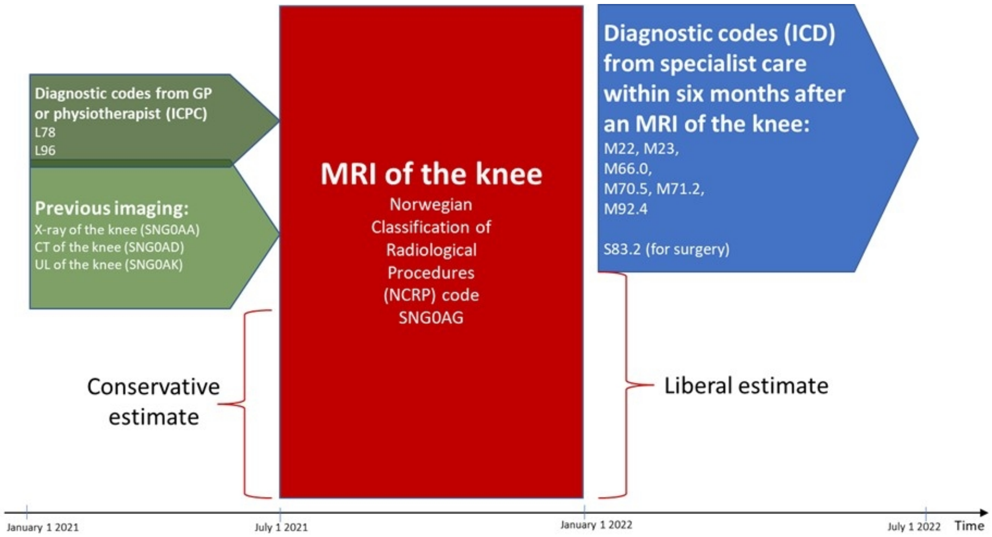

Figure 1 Illustration of the logic model of the data retrieval and indication of how two of the estimates of the proportion of low value imaging were calculated.

155x83mm (150 x 150 DPI)

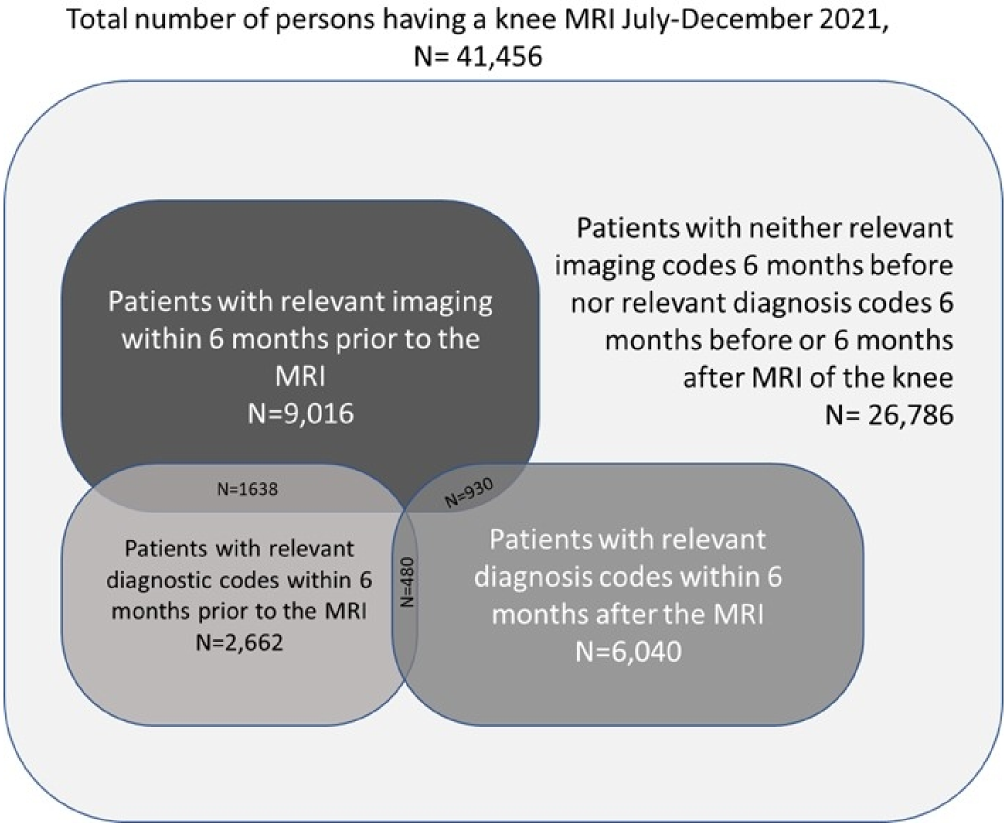

Figure 2 Illustration of the relationship between the various results

119x99mm (150 x 150 DPI)

Queries made by Department for Medical Reimbursement, Norwegian Directorate of Health

Lists pasientID and dates for MR Kne, point 1

```
select e.pasient_identifikasjon, to_char(e.datotid, 'YYYY-MM-DD') as dato
from odb_ncrpkode partition (y2021) n, odb_enkeltregning partition (y2021) e, odb_innsending i
where i.fagomraade_kode IN ('PO','LR') and
N.undersokelseskode in ('SNG0AG') and
i.innsending_status_k = 'godkjent' and
e.enkeltregning_status_k in ('godkjent', 'manuelt_godkjent') and
e.datotid >= '01.07.2021' and
e.datotid < '01.01.2022' and
n.enkeltregning_id = e.enkeltregning_id and
i.innsending_id = e.innsending_id
group by e.pasient_identifikasjon, to_char(e.datotid, 'YYYY-MM-DD')
order by 1,2;
```

Lists patients registered with selected diagnosis the last six moths after first MRI of the knee (SNG0AG), and removes the details to list relevant patients

```
select r.pasient_identifikasjon, r.forste_behandling, d.diagnose_kode, to_char(MIN(e.datotid),'dd.mm.yyyy') as
dato_diagnose from
(
select e.pasient_identifikasjon, MIN(e.datotid) as forste_behandling
from odb_ncrpkode partition (y2021) n, odb_enkeltregning partition (y2021) e, odb_innsending i
where 1=1
and i.innsending_id = e.innsending_id
and n.enkeltregning_id = e.enkeltregning_id
and i.fagomraade_kode IN ('PO','LR')
and n.undersokelseskode in ('SNG0AG')
and e.enkeltregning_status_k in ('godkjent', 'manuelt_godkjent')
and e.datotid >= '01.07.2021'
and e.datotid < '01.01.2022'
group by e.pasient_identifikasjon
) r, odb_enkeltregning e, odb_diagnose d, odb_innsending i where 1=1
and e.innsending_id = i.innsending_id
and e.enkeltregning_id = d.enkeltregning_id
and r.pasient_identifikasjon = e.pasient_identifikasjon
and i.fagomraade_kode IN('LE','PO')
and e.datotid > r.forste_behandling
and e.datotid < add_months(r.forste_behandling,6)
and d.diagnose_kode
IN('M220','M221','M222','M223','M224','M228','M229','M230','M231','M232','M233','M234','M235','M236','M238','M2
39','M660','M705','M712','M924','S832')
group by r.pasient_identifikasjon, r.forste_behandling, d.diagnose_kode;
```

Lists patients registered with specific diagnosis the first six months after the MRI of the knee – point 2 shows public/private distribution

```
select r.pasient_identifikasjon, r.fagomraade_kode from
(
select e.pasient_identifikasjon, i.fagomraade_kode, MIN(e.datotid) as forste_behandling
from odb_ncrpkode partition (y2021) n, odb_enkeltregning partition (y2021) e, odb_innsending i
where 1=1
and i.innsending_id = e.innsending_id
and n.enkeltregning_id = e.enkeltregning_id
and i.fagomraade_kode IN ('PO','LR')
and n.undersokelseskode in ('SNG0AG')
and e.enkeltregning_status_k in ('godkjent', 'manuelt_godkjent')
and e.datotid >= '01.07.2021'
and e.datotid < '01.01.2022'
group by e.pasient_identifikasjon, i.fagomraade_kode
) r, odb_enkeltregning e, odb_diagnose d, odb_innsending i where 1=1
and e.innsending_id = i.innsending_id
and e.enkeltregning_id = d.enkeltregning_id
and r.pasient_identifikasjon = e.pasient_identifikasjon
and i.fagomraade_kode IN('LE','PO')
and e.datotid > r.forste_behandling
and e.datotid < add_months(r.forste_behandling,6)
```

For peer review only

```
and d.diagnose_kode
IN('M220','M221','M222','M223','M224','M228','M229','M230','M231','M232','M233','M234','M235','M236','M238','M2
39','M660','M705','M712','M924','S832')
group by r.pasient_identifikasjon, r.fagomraade_kode;
```

Lists patients registered with specified diagnosis the six first months after MRI – point 3 shows shows those registered with a doctor/physiotherapist 6 months before the first SNG0AG, remove the details of the number of patients

```
select r.pasient_identifikasjon from
(
select e.pasient_identifikasjon, MIN(e.datotid) as forste_behandling
from odb_ncrpkode partition (y2021) n, odb_enkeltregning partition (y2021) e, odb_innsending i
where 1=1
and i.innsending_id = e.innsending_id
and n.enkeltregning_id = e.enkeltregning_id
and i.fagomraade_kode IN ('PO','LR')
and n.undersokelseskode in ('SNG0AG')
and e.enkeltregning_status_k in ('godkjent', 'manuelt_godkjent')
and e.datotid >= '01.07.2021'
and e.datotid < '01.01.2022'
group by e.pasient_identifikasjon
) r, odb_enkeltregning e, odb_diagnose d, odb_innsending i where 1=1
and e.innsending_id = i.innsending_id
and e.enkeltregning_id = d.enkeltregning_id
and r.pasient_identifikasjon = e.pasient_identifikasjon
and i.fagomraade_kode IN('LE')
and i.praksis_refusjonsgrunnlag in ('Fastlege','Fastlønnet','Turnuslege fastlønnet')
--and i.fagomraade_kode IN('LE','FY')
--and i.praksis_refusjonsgrunnlag not in ('Legevakt','Legevakt kommunal')
and e.datotid < r.forste_behandling
and e.datotid > add_months(r.forste_behandling,-6)
and d.diagnose_tabell in ('ICPC-2','ICPC-2B')
and (d.diagnose_kode like ('L78%') or
d.diagnose_kode like ('L96%'))
group by r.pasient_identifikasjon;group by r.pasient_identifikasjon, r.forste_behandling, d.diagnose_kode;
```

Lists the patients who are registered with SNG0AG, but who are not registered with any of the selected diagnostic codes six months before the first date with SNG0AG, point 3

```
select r.pasient_identifikasjon, r.forste_behandling from
(
select e.pasient_identifikasjon, MIN(e.datotid) as forste_behandling
from odb_ncrpkode partition (y2021) n, odb_enkeltregning partition (y2021) e
--, odb_innsending i
where 1=1
--and i.innsending_id = e.innsending_id
and n.enkeltregning_id = e.enkeltregning_id
--and i.fagomraade_kode IN ('PO','LR')
and e.enkeltregning_status_k in ('godkjent', 'manuelt_godkjent')
and n.undersokelseskode in ('SNG0AG')
and e.datotid >= '01.07.2021'
and e.datotid < '01.01.2022'
group by e.pasient_identifikasjon
) r where 1=1
and not exists(
select 0 from odb_enkeltregning partition (y2021) e, odb_diagnose partition (y2021) d, odb_innsending i where
1=1
and e.innsending_id = i.innsending_id
and e.enkeltregning_id = d.enkeltregning_id
and r.pasient_identifikasjon = e.pasient_identifikasjon
and i.fagomraade_kode IN('LE')
and i.praksis_refusjonsgrunnlag in ('Fastlege','Fastlønnet','Turnuslege fastlønnet')
--and i.fagomraade_kode IN('LE','FY')
--and i.praksis_refusjonsgrunnlag not in ('Legevakt','Legevakt kommunal')
and e.datotid < r.forste_behandling
and e.datotid > add_months(r.forste_behandling,-6)
and d.diagnose_tabell in ('ICPC-2','ICPC-2B')
and (d.diagnose_kode like ('L78%') or d.diagnose_kode like ('L96%'))
```

For peer review only

```
);
```

Lists patients registered with SNG0AG and registered with selected NCRP codes up to six months before the first date with SNG0AG, point 4

```
select r.pasient_identifikasjon from
(
select e.pasient_identifikasjon, MIN(e.datotid) as forste_behandling
from odb_ncrpkode partition (y2021) n, odb_enkeltregning partition (y2021) e, odb_innsending i
where 1=1
and i.innsending_id = e.innsending_id
and n.enkeltregning_id = e.enkeltregning_id
and i.fagomraade_kode IN ('PO','LR')
and n.undersokelseskode in ('SNG0AG')
and e.enkeltregning_status_k in ('godkjent', 'manuelt_godkjent')
and e.datotid >= '01.07.2021'
and e.datotid < '01.01.2022'
group by e.pasient_identifikasjon
) r, odb_enkeltregning e, odb_ncrpkode partition (y2021) n, odb_innsending i where 1=1
and e.innsending_id = i.innsending_id
and e.enkeltregning_id = n.enkeltregning_id
and r.pasient_identifikasjon = e.pasient_identifikasjon
and i.fagomraade_kode IN('PO','LR')
and e.datotid < r.forste_behandling
and e.datotid > add_months(r.forste_behandling,-6)
and n.undersokelseskode in ('SNG0AA','SNG0AD','SNG0AK')
group by r.pasient_identifikasjon;
```

How many in the sample above are not included in the UU1 sample, item 4

```
select count (distinct p.fnr)
from TMP_VH_23_HOFMANN_SML_MED_UU1 p where 1=1
and not exists(select 0 from TMP_VH_23_HOFMANN_UU1 r where 1=1
and p.fnr = r.fnr);
```

How many in the main sample have M17, but not SNG0AA

```
select r.pasient_identifikasjon from
(
select e.pasient_identifikasjon, i.fagomraade_kode, MIN(e.datotid) as forste_behandling
from odb_ncrpkode partition(y2021) n, odb_enkeltregning partition(y2021) e, odb_innsending i
where 1=1
and i.innsending_id = e.innsending_id
and n.enkeltregning_id = e.enkeltregning_id
and i.fagomraade_kode IN ('PO','LR')
and n.undersokelseskode in ('SNG0AG')
and e.enkeltregning_status_k in ('godkjent', 'manuelt_godkjent')
and e.datotid >= '01.07.2021'
and e.datotid < '01.01.2022'
group by e.pasient_identifikasjon, i.fagomraade_kode
) r, odb_enkeltregning partition(y2021) e, odb_diagnose partition(y2021) d, odb_innsending i where 1=1
and e.innsending_id = i.innsending_id
and e.enkeltregning_id = d.enkeltregning_id
and r.pasient_identifikasjon = e.pasient_identifikasjon
and i.fagomraade_kode IN('LE','PO')
and e.datotid > r.forste_behandling
and e.datotid < add_months(r.forste_behandling,6)
and d.diagnose_kode like('M17%')
and not exists(select 0 from odb_ncrpkode partition(y2021) n2, odb_enkeltregning partition(y2021) e2 where 1=1
and n2.enkeltregning_id = e2.enkeltregning_id
and e2.pasient_identifikasjon = r.pasient_identifikasjon
and n2.undersokelseskode in ('SNG0AA')
and e2.datotid < r.forste_behandling
and e2.datotid > add_months(r.forste_behandling,-6)
)
group by r.pasient_identifikasjon;
```

The RECORD statement – checklist of items, extended from the STROBE statement, that should be reported in observational studies using routinely collected health data.

	Item No.	STROBE items	Location in manuscript where items are reported	RECORD items	Location in manuscript where items are reported
**Title and abstract**					
	1	Low value MRI of the knee in Norway<br>A register-based study to identify the proportion of low-value MRIs and estimate the related costs	Title and abstract	RECORD 1.1: Register study based on conditional data extraction and analysis of data from Control and Reimbursement of Healthcare Claims (CRHC) registry in Norway.<br><br>RECORD 1.2: Norway<br><br>RECORD 1.3: conditional data extraction and analysis of data from Control and Reimbursement of Healthcare Claims (CRHC) registry in Norway.	1
**Introduction**					
Background rationale	2	Explain the scientific background and rationale for the investigation being reported	Introduction	Uncritical and inappropriate use of radiology may reduce quality and safety of care, e.g., in terms of false (positive and negative) results, misdiagnosis, incidental findings of uncertain significance, overdiagnosis, delays in appropriate care, and increased costs. Accordingly, it is crucial to reduce the use of low-value examinations, i.e., examinations that do not lead to change in patient management or are deemed not to be beneficial.	1
Objectives	3	State specific objectives, including any prespecified hypotheses	Introduction	The objective of this study is to estimate the proportion of examinations that are unlikely to have	1

For peer review only

				benefited patients and that potentially are of low value and to provide an estimate of the related costs.	
**Methods**					
Study Design	4	Present key elements of study design early in the paper		The study uses register data to identify patients that did not have relevant diagnoses or actions six months before or after an MRI of the knee	1
Setting	5	Describe the setting, locations, and relevant dates, including periods of recruitment, exposure, follow-up, and data collection		In Norway, all residents are entitled to essential medical care,21 and due to universal health coverage, most health services are publicly funded through taxes.22 Specialists and radiological services are organized within the specialist care, governed by the regional hospital trusts.23 Physiotherapist and general practitioners (GP) are organized in the primary care which are the municipalities' responsibility. However, several orthopedist and physiotherapist clinics operate in the private health care sector either on contracts with the public health system or independently.24 Private imaging centers perform about 20-25% of all outpatient examinations, where 60% of these are Computed Tomography (CT) or MRI-examinations.25 A significant portion of these examinations is commissioned by public health services to ease the pressure on public services.23 Further, private imaging centers offer insurance or out-of-pocket financed radiology, which allows shorter waiting times for patients. About 10 percent of the	2

For peer review only

				population have private health insurances.22	
Participants	6	*Register study:*	Data provided by the Control and Reimbursement of Healthcare Claims (CRHC) registry by the Norwegian Directorate of Health. Specified types of registry data were retrieved for all patients that performed an outpatient knee MRI at public hospitals and/or private imaging centers during a six-month period (July 1 to December 31, 2021).	RECORD 6.1: The methods of study population selection (such as codes or algorithms used to identify subjects) should be listed in detail. If this is not possible, an explanation should be provided.<br><br>RECORD 6.2: Any validation studies of the codes or algorithms used to select the population should be referenced. If validation was conducted for this study and not published elsewhere, detailed methods and results should be provided.<br><br>RECORD 6.3: If the study involved linkage of databases, consider use of a flow diagram or other graphical display to demonstrate the data linkage process, including the number of individuals with linked data at each stage.	3
Variables	7	Clearly define all outcomes, exposures, predictors, potential confounders, and effect modifiers. Give diagnostic criteria, if applicable.		RECORD 7.1: A complete list of codes and algorithms used to classify exposures, outcomes, confounders, and effect modifiers is provided in a supplementary file.	Page 4 and Supplement
Data sources/ measurement	8	For each variable of interest, give sources of data and details of methods of assessment (measurement).<br>Describe comparability of assessment methods if there is more than one group		Data from Control and Reimbursement of Healthcare Claims (CRHC) registry by the Norwegian Directorate of Health. Specified types of registry data were retrieved for all patients that performed an outpatient knee MRI at public hospitals and/or private imaging centers during a six-month period (July 1 to December 31, 2021).	

For peer review only

Bias	9	Describe any efforts to address potential sources of bias		Coding errors.	6-8
Study size	10	Explain how the study size was arrived at		All outpatient MRIs of the knee in the whole country are included	3
Quantitative variables	11	Explain how quantitative variables were handled in the analyses. If applicable, describe which groupings were chosen, and why		See table and Figure 1 and 2	3,5
Statistical methods	12	(a) Describe all statistical methods, including those used to control for confounding<br>(b) Describe any methods used to examine subgroups and interactions<br>(c) Explain how missing data were addressed<br>(d) *Cohort study* - If applicable, explain how loss to follow-up was addressed<br>*Case-control study* - If applicable, explain how matching of cases and controls was addressed<br>*Cross-sectional study* - If applicable, describe analytical methods taking account of sampling strategy<br>(e) Describe any sensitivity analyses		No statistical methods were applied	
Data access and cleaning methods		..		RECORD 12.1: Authors should describe the extent to which the investigators had access to the database population used to create the study population.<br>One of the co-authors has full access to all data and the others have full access to aggregate data.	2

For peer review only

				RECORD 12.2: Authors should provide information on the data cleaning methods used in the study. Data in public records are precleaned.	
Linkage		..		RECORD 12.3: State whether the study included person-level, institutional-level, or other data linkage across two or more databases. The methods of linkage and methods of linkage quality evaluation should be provided. Conditional retrieval. Hence, internal linkage.	2-3
**Results**					
Participants	13	(a) Report the numbers of individuals at each stage of the study (*e.g.*, numbers potentially eligible, examined for eligibility, confirmed eligible, included in the study, completing follow-up, and analysed)<br>(b) Give reasons for non-participation at each stage.<br>(c) Consider use of a flow diagram	A total number of 48,212 MRIs were conducted during the period, divided by 41,456 unique patients and 45,946 reimbursement claims (used for categorizing reimbursements from CRHC).	RECORD 13.1: Describe in detail the selection of the persons included in the study (*i.e.,* study population selection) including filtering based on data quality, data availability and linkage. The selection of included persons can be described in the text and/or by means of the study flow diagram.	4
Descriptive data	14	(a) Give characteristics of study participants (*e.g.*, demographic, clinical, social) and information on exposures and potential confounders<br>(b) Indicate the number of participants with missing data for each variable of interest<br>(c) *Cohort study* - summarise follow-up time (*e.g.*, average and total amount)	MRIs of the knee from the whole country.		

For peer review only

Outcome data	15	*Cohort study* - Report numbers of outcome events or summary measures over time<br>*Case-control study* - Report numbers in each exposure category, or summary measures of exposure<br>*Cross-sectional study* - Report numbers of outcome events or summary measures			
Main results	16	(a) Give unadjusted estimates and, if applicable, confounder-adjusted estimates and their precision (e.g., 95% confidence interval). Make clear which confounders were adjusted for and why they were included<br>(b) Report category boundaries when continuous variables were categorized<br>(c) If relevant, consider translating estimates of relative risk into absolute risk for a meaningful time period			
Other analyses	17	Report other analyses done—e.g., analyses of subgroups and interactions, and sensitivity analyses			
**Discussion**					
Key results	18	Summarise key results with reference to study objectives			4-5
Limitations	19	Discuss limitations of the study, taking into account sources of potential bias or imprecision. Discuss both direction and magnitude of any potential bias		RECORD 19.1: Discuss the implications of using data that were not created or collected to answer the specific research question(s). Include discussion of misclassification bias, unmeasured confounding, missing data, and changing eligibility over	4-5

For peer review only

				time, as they pertain to the study being reported.	
Interpretation	20	Give a cautious overall interpretation of results considering objectives, limitations, multiplicity of analyses, results from similar studies, and other relevant evidence			5
Generalisability	21	Discuss the generalisability (external validity) of the study results		No claims about generalizability as study includes all examinations.	
**Other Information**					
Funding	22	Give the source of funding and the role of the funders for the present study and, if applicable, for the original study on which the present article is based		Research Council of Norway (Project number 302503)	8
Accessibility of protocol, raw data, and programming code		..		RECORD 22.1: Authors should provide information on how to access any supplemental information such as the study protocol, raw data, or programming code.	Supplementary File

*Reference: Benchimol EI, Smeeth L, Guttmann A, Harron K, Moher D, Petersen I, Sørensen HT, von Elm E, Langan SM, the RECORD Working Committee. The REporting of studies Conducted using Observational Routinely-collected health Data (RECORD) Statement. *PLoS Medicine* 2015; in press.

*Checklist is protected under Creative Commons Attribution (CC BY) license.
